# Melanocortin 1 receptor activation protects against alpha-synuclein pathologies in models of Parkinson’s disease

**DOI:** 10.1186/s13024-022-00520-4

**Published:** 2022-02-23

**Authors:** Waijiao Cai, Pranay Srivastava, Danielle Feng, Yue Lin, Charles R. Vanderburg, Yuehang Xu, Pamela Mclean, Matthew P. Frosch, David E. Fisher, Michael A. Schwarzschild, Xiqun Chen

**Affiliations:** 1 MassGeneral Institute for Neurodegenerative Disease, Department of Neurology, Massachusetts General Hospital, Harvard Medical School, Boston, USA; 2 Department of Integrative Medicine, HuaShan Hospital, Institutes of Integrative Medicine, Fudan University, Shanghai, China; 3 Aligning Science Across Parkinson’s (ASAP) Collaborative Research Network, Chevy Chase, Towson, MD USA; 4 Harvard NeuroDiscovery Advanced Tissue Resource Center, Massachusetts General Hospital, Harvard Medical School, Boston, USA; 5 Mayo Clinic, Jacksonville, FL USA; 6 Neuropathology Service, Massachusetts General Hospital, Harvard Medical School, Boston, USA; 7 Cutaneous Biology Research Center, Department of Dermatology, Massachusetts General Hospital, Harvard Medical School, Boston, USA

**Keywords:** Melanocortin 1 receptor, Alpha-synuclein, Parkinson’s disease, Melanoma, Nuclear factor erythroid 2-related factor 2

## Abstract

**Background:**

Epidemiological studies suggest a link between the melanoma-related pigmentation gene melanocortin 1 receptor (*MC1R*) and risk of Parkinson’s disease (PD). We previously showed that MC1R signaling can facilitate nigrostriatal dopaminergic neuron survival. The present study investigates the neuroprotective potential of MC1R against neurotoxicity induced by alpha-synuclein (αSyn), a key player in PD genetics and pathogenesis.

**Methods:**

Nigral dopaminergic neuron toxicity induced by local overexpression of aSyn was assessed in mice that have an inactivating mutation of *MC1R*, overexpress its wild-type transgene, or were treated with MC1R agonists. The role of nuclear factor erythroid 2-related factor 2 (Nrf2) in MC1R-mediated protection against αSyn was characterized in vitro. Furthermore, MC1R expression was determined in human postmortem midbrain from patients with PD and unaffected subjects.

**Results:**

Targeted expression of αSyn in the nigrostriatal pathway induced exacerbated synuclein pathologies in *MC1R* mutant mice, which were accompanied by neuroinflammation and altered Nrf2 responses, and reversed by the human *MC1R* transgene. Two MC1R agonists were neuroprotective against αSyn-induced dopaminergic neurotoxicity. In vitro experiments showed that Nrf2 was a necessary mediator of MC1R effects. Lastly, MC1R was present in dopaminergic neurons in the human substantia nigra and appeared to be reduced at the tissue level in PD patients.

**Conclusion:**

Our study supports an interaction between MC1R and αSyn that can be mediated by neuronal MC1R possibly through Nrf2. It provides evidence for MC1R as a therapeutic target and a rationale for development of MC1R-activating strategies for PD.

**Supplementary Information:**

The online version contains supplementary material available at 10.1186/s13024-022-00520-4.

## Background

Parkinson’s disease (PD) is a common neurodegenerative disorder and a leading cause of long-term disability. Although symptomatic treatments are available and effective, at least partially, there is currently no therapy known to reverse, arrest, or slow its progressive course. Multiple genetic and environmental factors contribute to the development of PD; among them, alpha-synuclein (αSyn, encoded by *SNCA*) plays a central role in PD genetics and pathogenesis [1, 2]. Mutations in *SNCA* can cause PD, and accumulation and aggregation of αSyn within Lewy bodies and Lewy neurites in the nervous system are a pathological hallmark of PD. Various cellular events including proteinopathy, neuroinflammation, and oxidative stress contribute to the degenerative process, leading to the eventual loss of dopaminergic neurons of the nigrostriatal dopaminergic pathway of the brain, another pathological hallmark of PD [3]. As proteostasis, the redox system, and inflammatory processes in PD can be orchestrated by the nuclear factor erythroid 2-related factor 2 (Nrf2), activation of Nrf2 is a promising therapeutic approach for neurodegenerative disease [4, 5].

Melanocortin 1 receptor (*MC1R*) is the major genetic determinant of hair color. Binding of its ligand alpha-melanocyte stimulating hormone (α-MSH) to MC1R on melanocytes activates the cAMP pathway and facilitates brown/black eumelanin synthesis and increases the ratio of yellow/red pheomelanin to eumelanin [6, 7]. Severe loss-of-function polymorphisms of *MC1R* contributes to red hair/fair skin and are associated with skin aging, and melanoma risk [8–10]. More recent studies identify a critical role of MC1R in regulating physiological functions in the skin including the immune response, DNA repair, and cell differentiation and proliferation, which can be pigmentation-dependent or -independent [10, 11]. In addition to its cutaneous expression and function, MC1R is expressed in other tissue and cell types, including immune and endothelial cells, and can modulate the immune system and inflammatory response [12, 13]. α-MSH or its synthetic analog Nle^4^,D-Phe^7^-α-MSH (NDP-MSH), a tanning agent and drug approved by the European Medicines Agency for treating the photosensitive skin condition erythropoietic porphyria [14], exerts protective effects in models of ischemic stroke, traumatic brain injury, spinal cord injury, Alzheimer’s disease, and neuroinflammatory disease [15–18], with MC1R engagement shown to mediate this protection in the latter model.

Prompted by well-documented epidemiological associations between MC1R, pigmentation, and melanoma, and between melanoma and PD as well as possible associations between red hair and PD, and between MC1R variants and PD [19], we previously demonstrated the presence of MC1R in dopaminergic neurons in the mouse substantia nigra (SN) and its influence on dopaminergic neuron survival [20]. Here, we report MC1R-specific protection against αSyn oligomerization and related inflammation and dopaminergic neurotoxicity. In vitro analyses revealed that MC1R counteracted αSyn oligomerization by activating Nrf2. Further, we demonstrate that PD patients exhibited reduced levels of MC1R in the SN.

## Methods

### Study design

The objective of this study was to characterize the protective role of MC1R in the nigrostriatal dopaminergic pathway and to elucidate responsible downstream mediators. Complementary genetic and pharmacological approaches were employed to manipulate MC1R in vivo and in vitro. αSyn pathologies, neuroinflammation, and related dopaminergic neurotoxicity were induced by overexpressing human wild-type (WT) αSyn in mice and in HEK cells and primary neuronal cultures. Human studies entailed assessment of postmortem brain tissue from PD patients and controls.

Sample sizes for animal experiments were determined based on our previous studies [20, 21] in which significant differences in primary outcome measures (nigral dopaminergic cell counts and striatal dopamine content) were observed. For all animal experiments involving genetic modification of *MC1R* and quantification of the outcome measures, littermates were used as controls. For animal experiments using commercially obtained mice, grouping was randomized. Cell experiments were repeated at least three times with at least three replicates within each condition. Investigators were blind to treatment assignments and/or sample group information wherever practical. All animal and human study protocols were approved by the responsible authorities at Massachusetts General Hospital.

### Experimental animals

*MC1R* extension (*MC1R*^e/e^) mice carrying an inactivating frameshift mutation of *MC1R* in a C57BL/6 J background [20, 22] were backcrossed with C57BL/6 J mice from the Jackson Laboratory (Bar Harbor, ME). Offspring heterozygous breeders were crossed with each other to generate *MC1R*^e/e^ and littermate WT mice.

*MC1R* transgenic (Tg) mice in an e/e background (*MC1R*^e/e^Tg) were originally generated and characterized at University of Edinburgh, UK [23]. *MC1R*^e/e^Tg mice express the human *MC1R* under the transcriptional control of its human promoter, yielding a physiological expression pattern similar to that in humans. The transgene rescues the *MC1R* deficiency dermal phenotype to give *MC1R*^e/e^Tg mice a WT-like dark coat. *MC1R*^e/e^Tg mice were crossed with *MC1R*^e/e^ mice to generate *MC1R*^e/e^Tg and littermate *MC1R*^e/e^ mice.

To test the effects of the MC1R agonist BMS-470539, 3-month-old male C57Bl/6 J mice were purchased from the Jackson Laboratory. To test the effects of the MC1R agonist NDP-MSH, *MC1R*^e/e^ mice and their WT littermates were used.

Mice were maintained in home cages at a constant temperature with a 12-h light/dark cycle and free access to food and water.

### Viral vectors and intra-SN infusion

Vector production and stereotaxic virus intra-SN infusion were described previously [24]. The vectors used were: (1) p adeno-associated virus (AAV)-CBA-human αSyn-WPRE (αSyn AAV), (2) pAAV-CBA-WPRE empty vector (vector), (3) pAAV-CBA-venus1-human αSyn-WPRE and pAAV-CBA-human αSyn-venus2-WPRE bimolecular fluorescence complementation (BiFC αSyn AAV), and (4) pAAV-CBA-Venus-WPRE (venus).

Viral vectors were infused at a volume of 2 µl into the left SN at the following coordinates: AP + 0.9 mm, ML + 1.2 mm, and DV -4.3 mm relative to lambda.

### MC1R agonist treatments

NDP-MSH and BMS‐470,539 dihydrochloride were purchased from Tocris Bioscience (Bristol, UK). BMS-470539 (20 mg/kg) or vehicle saline was administered subcutaneously daily starting 1 day after αSyn AAV or empty vector infusion for 4 weeks. A total dose of 3 nmol NDP-MSH in 2 µl PBS was injected intracranially at 30 µl/60 min into the left striatum (coordinates: AP + 0.9 mm, ML + 2.2 mm, and DV -2.5 mm relative to bregma). Control mice received PBS injection. αSyn AAV was infused into the SN immediately after NDP-MSH or vehicle administration.

### Sequential tissue extraction and αSyn immunoblotting

Mice were sacrificed, and their ventral midbrain and striatum were dissected. Protein sequential extraction and immunoblotting of αSyn were conducted as previously reported [24] with modifications. Briefly, tissues were homogenized in 1% Triton X-100 buffer and centrifuged. The supernatant was designated as the “Triton X-100-soluble” fraction. The pellet was resuspended in lysis buffer containing 2% SDS and designated as the “SDS-soluble” fraction. Protein concentrations were determined by BCA protein assay. Protein from each Triton X-100-soluble (50 µg) and SDS-soluble (80 µg) sample were run on NuPAGE 4–12% SDS-PAGE gel and transferred to PVDF membranes following fixation with 0.4% paraformaldehyde for 30 min. Primary antibody against human αSyn (clone Syn211, ThermoFisher Scientific, AHB0261) was added at 1:700 and incubated overnight at 4 °C. Membranes were then incubated with a secondary antibody. Signals were detected using enhanced chemiluminescence. Band densities were determined using ImageJ and normalized to ponceau staining.

### Immunostaining, imaging, and quantification

Mice were sacrificed, and their brains were processed and sectioned coronally as described [24]. For immunostaining, sections were incubated with primary antibodies overnight at 4ºC and corresponding secondary antibodies for 1 h at 37 ºC. The primary antibodies used were against human αSyn (clone Syn211, ThermoFisher Scientific, AHB0261) at 1:500, phosphorylated αSyn at serine 129 (p-αSyn) (p-syn/81A, BioLegend, 825,701) at 1:500, glial fibrillary acidic protein (GFAP) (clone GA5, Sigma, G3893 and MAB360) at 1:1000, ionized calcium binding adapter molecule 1 (iba1) (clone EPR16588, Abcam, ab178846, and ab107159) at 1:500, tyrosine hydroxylase (TH) (clone TH2, Sigma, T1299) at 1:1000, and Nrf2 (Abcam, ab31163) at 1:500. For fluorescence staining, sections were incubated with goat anti-rabbit or anti-mouse lgG-Alexafluor-546 or -488. For DAB staining, sections were incubated with appropriate secondary antibodies, and the staining was developed by incubating with DAB.

MC1R staining was performed as previously reported [20] with modifications. Sections were heated for antigen retrieval and incubated with primary anti-MC1R (Santa Cruz, SC‐19,485) at 1:50 or anti-human MC1R (LSBio LS-A1040) at 1:100 overnight at 4 °C [25]. Sections were then incubated with Alexa Fluor conjugated secondary antibody at 1:200 at 37 ºC for 30 min. After washes, subsequent TH or GFAP or iba1 staining was performed.

Fluorescence images were captured under a Nikon C2s laser scanning microscope. Images from DAB-stained sections were captured under an Olympus BX50 microscope with a DP 70 digital camera system. Posterior (interaural 0.00/bregma -3.80 mm), posterior central (interaural 0.28 mm/bregma -3.52 mm), anterior central (interaural 0.64 mm/bregma -3.16 mm), and anterior (interaural 0.88 mm/bregma -2.92 mm) midbrain sections from each mouse [26] were selected for quantification unless stated otherwise.

To evaluate αSyn transduction efficiency, midbrain sections were co-labeled with antibodies against human αSyn and TH. To determine the percentage of αSyn-positive dopaminergic neurons in the SN, images were acquired in 488- and 546-nm channels with 40 × magnification. ImageJ software was used to count TH-positive cells and cells that were both TH- and αSyn-positive.

Quantification of p-αSyn staining was performed using the optical fractionator method at 40 × magnification (Olympus BX51 microscope and Olympus CAST stereology software) [27] to count positively stained particles in the SN.

For astrogliosis and microgliosis analyses, GFAP staining and the morphology of iba1-positive cells in the SN pars compacta (SNpc) were analyzed as previously described [24].

For proteinase K digestion, sections were mounted onto slides, dried overnight, and cover-slipped [24]. After image acquisition, cover slips were carefully removed, and sections were rehydrated. Sections were incubated with 50 μg/ml proteinase K at 55 °C for 120 min, and images were recaptured. Reconstituted venusYFP intensity was quantified using ImageJ.

For thioflavin-S staining and quantification, sections were incubated with 0.05% thioflavin S solution for 8 min [28]. Images were recaptured in 488-nm channel at 40 × magnifications. Thioflavin-S fluorescence intensity was quantified using ImageJ.

To assess nuclear-to-cytoplasmic Nrf2 ratio, sections were counterstained by DAPI to reveal the nucleus, and nuclear Nrf2 signal was defined within DAPI regions. Sections were imaged at excitations of 488 nm for Nrf2 and 568 nm for TH. The mean fluorescence intensities of nuclear and cytoplasmic Nrf2 per cell were measured, and the ratio was determined using ImageJ’s “Intensity Ratio Nuclei Cytoplasm Tool” as previously described [29]. Only TH-positive neurons were measured. Five to ten cells were randomly picked on each side of the SN from each section and a total of 30 cells on each side from each animal were analyzed.

### Protein oxidation

Protein carbonyls in ventral midbrain tissue were detected using an Oxyblot protein oxidation detection kit (Millipore, S7150) according to the manufacturer’s instructions. Band density was analyzed using ImageJ and normalized to ponceau staining density.

### Western blotting

Ventral midbrain tissues were lysed, and proteins were extracted and electrophoresed. The blot was probed with anti-Nrf2 (Abcam, ab31163) at 1:1000 or anti-MC1R (Santa Cruz, SC-19485) at 1:1000 or anti-TH (ENZO BML-SA497-0100) at 1:1000. Band density was analyzed using ImageJ and normalized by actin. All the original full-blot images are in Supplemental files, Fig. S6 and Fig. S7.

### Quantitative polymerase chain reaction (qPCR) for cytokines and Nrf2 target genes

Total RNA was isolated using TRI reagent (Invitrogen) and reverse-transcribed into cDNA using a superscript III kit (Invitrogen). qPCR was performed in a 96-well plate using SYBR Green PCR Master Mix in an Applied Biosystem 7500. GAPDH was used to normalize expression levels of the target genes. The 2^–∆∆Ct^ method was employed for data analysis [30]. The primers used are provided in Supplementary Table 1.

### Amphetamine-induced rotational behavior

Amphetamine-induced (5 mg/kg, intraperitoneal) rotational behavior was assessed by an automated rotometry system (San Diego Instruments) for 60 min as previously described [24, 31].

### Striatal dopamine measurement

Mice were sacrificed, and the striatum was dissected. Dopamine content was determined by high-performance liquid chromatography (HPLC) coupled with electrochemical detection as previously described [24, 31].

### Stereological analysis of SN dopaminergic neurons

A complete set of serial midbrain sections were collected and immunostained for TH and counterstained for Nissl to reveal dopaminergic neurons and total neurons. Unbiased stereological counting was performed as previously described [24, 31].

### HEK293T cell transfection, transduction, immunostaining, immunoblotting, and chromatin immunoprecipitation (ChIP)-qPCR

HEK293T cells were purchased from Clonetech. Cells were maintained in DMEM with 10% FBS in a humidified incubator at 37 °C with 5% CO_2_. pcDNA3.1( +)-human WT αSyn and control plasmid were provided by a former colleague Dr. Joseph Mazzulli (Northwestern University, IL) [32]. MC1R-Tango expressing human MC1R tagged with FLAG and vector control GPRC5A-Tango were gifts from Bryan Roth (Addgene plasmid #66,427 and #66,382). αSyn and MC1R and their respective controls were transfected into cells using Lipofectamine® 2000 (ThermoFisher Scientific) according to the manufacturer’s instructions.

Human shNrf2 was purchased from Dharmacon RNAi Consortium (RHS4533-EG4780). pLKO.1- scrambled RNA (scRNA) (Sigma, SHC016-1EA) was used as a control. Plasmids were packaged in lentivirus with packaging plasmid psPAX2 and envelope plasmid pMD2.G (Addgene plasmid #12,259 and #12,260, gifts from Didier Trono). Lentiviral particles were produced in HEK293T cells. For scRNA or shNrf2 transduction, cells were incubated in medium containing lentiviral particles in the presence of polybrene for 16 h.

Cells were harvested 48 h after transfection with or without viral transduction and lysed for immunoblotting or qPCR. For αSyn immunoblotting, in-cell crosslinking was performed using disuccinimidyl suberate ligand (ThermoFisher Scientific) as previously described [33]. Primary antibodies used were anti-human αSyn (clone Syn211, ThermoFisher Scientific, AHB0261) at 1:500, anti-Nrf2 (Abcam, ab31163) at 1:1000, anti-FLAG (Sigma, F1804) at 1:1000, anti-phosphorylated cAMP response element-binding protein (pCREB) (Ser133) (Cell Signaling, 9198S) at 1:1000, anti-CREB binding protein (CBP) (Cell Signaling, 7389S) at 1:1000, and anti- MC1R (LSBio LS-A1040) at 1:1000. Densities of bands were analyzed using ImageJ and normalized by actin.

For chromatin immunoprecipitation (ChIP)-qPCR assay [34, 35] identifying CREB binding to *Nrf2* following MC1R activation, cells (5 × 10^6^) were transfected with αSyn and MC1R and harvested 48 h after transfection. ChIP was performed using the ChIP-IT® Express (Active Motif, 53,008) following the manufacturer’s instructions [36]. In brief, cells were fixed with 37% formaldehyde (12 min) followed by sonication (25% amplitude, pulse for 30 s on and 30 s off for a total of 15 cycles). Immunoprecipitation was carried out using antibodies against anti-pCREB (Ser 133) (Cell Signaling, 9198S) or nonspecific rabbit IgG (Cell Signaling, 2729S) as negative control. Following antibody pull-down and DNA purification, qPCR was conducted. Forward and reverse primers used were CGGGCTGAGCTTCCGAAAAT and AACTCTTTATCTCGCGGGCG. The primers were designed to detect CREB binding site TGACG in the *Nrf2* promoter based on published information [37, 38], which also matched the predicted CREB binding site from our own in silico promoter analysis of *Nrf2*. The program for quantification amplification was 2 min at 95 °C, 15 s at 95 °C, 20 s at 58 °C and 20 s at 72 °C for 40 cycles in 20 μl reaction volume. Data was presented as % input genomic DNA for different treatment groups.

For immunocytochemistry, cells were grown on pre-coated poly-lysine coverslips, fixed 48 h post-transfection with or without viral transduction in 4% PFA for 10 min, and blocked in 5% normal goat serum in PBS/0.3% Triton X-100 for 30 min at 37 ºC. Cells were incubated with anti-Nrf2 (Abcam, ab31163, 1:200) overnight at 4ºC and Alexa-Fluor-488 for 1 h at 37 ºC. Images were acquired under a Nikon C2s laser scanning microscope. The subcellular distribution of Nrf2 fluorescence in nuclear and cytoplasmic regions was quantified using ImageJ as described above and previously in cell cultures [29].

### Primary neuron cultures, viral transduction, NDP-MSH treatment, immunostaining and quantification, and cell assays

Primary cortical neurons were prepared as previously described [39, 40] from the cerebral cortex of embryonic day 16–17 WT and *MC1R*^e/e^ mice. Mouse shNrf2 was purchased from Sigma (SHCLNG, NM 010,902) and packaged with lentivirus. For lentiviral transduction, cells were incubated in 250 μl medium containing lentiviral scRNA or shNrf2 and 250 μl complete neuron growth medium (Neurobasal medium with 2% B27 supplement, 2 mM L-glutamine, 100 U/ml penicillin, and 100 µg/ml streptomycin) in the presence of polybrene at DIV3 for 48 h in a 24-well plate. BiFC αSyn AVV or control venus AAV was added at 1.25 × 10^10^ gc to each well for 48 h. Medium was changed to complete neuron growth medium supplemented with or without 10 nM NDP-MSH at DIV5. At DIV12, 50 μl supernatant from each well was taken for lactate dehydrogenase (LDH) release assay using a Pierce LDH Cytotoxicity Assay Kit (ThermoFisher Scientific 88,953, 88,954) according to the manufacturer’s instructions. Nrf2 knock-down efficiency was determined by qPCR.

At DIV9, images of living neurons were obtained under an inverted Leica fluorescence microscope. At DIV12, neurons transduced with αSyn AAV or control venus AAV were fixed and immunostained. Primary antibodies used were against MAP2 (Invitrogen, PA5-17,646, or 13–1500 for double staining) at 1:250, MC1R (Santa Cruz, SC‐19,485) at 1:1000, and human αSyn (Thermo Fisher Scientific, AHB0261) at 1:250. Cell were counterstained by DAPI.

For MAP2-positive cell counting, three images were captured randomly from each coverslip under a 20 × objective in an Olympus BX50 microscope equipped with a DP70 digital camera system. MAP2 and DAPI channels were merged, and MAP2-positive cells in each visual field were counted using ImageJ [41]. A total of nine images from three replicate coverslips were analyzed.

### Human samples

Postmortem frozen SN tissue from pathologically diagnosed PD patients and control individuals with no neurological conditions was obtained from the Massachusetts Alzheimer’s Disease Research Center of the Massachusetts General Hospital. Age and postmortem interval (PMI) for cases used for each of the experiments were provided in figure legends. All subjects were Caucasian. Tissues were cryosectioned at 8 μm and airdried at 37 ºC for 2 min and fixed in 70% ethanol for 2 min. For peroxidase immunostaining, sections were immersed in 1% H_2_O_2_ in 100% methanol for 10 min at 37 ºC to quench endogenous peroxidase. Sections were then blocked by 5% normal goat serum and incubated with anti-human MC1R (LSBio, LS-A1040) at 1:50 and/or anti-TH (Sigma, T1299) at 1:500 at 37 ºC for 45 min. After three washes, sections were incubated with either biotin-conjugated secondary antibody at 1:500 for peroxidase immunostaining or Alexa Fluor-conjugated secondary antibodies at 1:200 for fluorescent immunostaining at 37 ºC for 30 min.

For immunoblotting, twenty Sects. (10 μm) containing only SN from frozen tissues were collected in RIPA lysis buffer. Protein sample (120 µg) was loaded and run on NuPAGE 4–12% SDS-PAGE gels at 150 voltage for 80 min and transferred to PVDF membranes at 90 voltage for 75 min at 4 ºC. Blots were then probed by anti-human MC1R (LSBio LS-A1040) at 1:700, anti TH (Sigma, T1299) at 1:1000, and anti Nrf2 (D1Z9C) (Cell Signaling 12721S) at 1:1000, overnight at 4 ºC, followed by an extra one-hr incubation at 37 ºC for MC1R probing to enhance the binding. Actin was used as the loading control. Blots were then incubated with anti-rabbit or anti-mouse secondary antibodies at 1: 1000 for 1 h at 37 ºC. Blots were incubated in enhanced chemiluminescence for 2 min and then scanned by an LI-COR Odyssey Fc Image System.

All human tissue study protocols were approved by the Partners Human Research Committee.

### Statistical analysis

All values are presented as the mean ± standard error of mean (SEM). The comparison between two groups was conducted using unpaired two-tail Student’s t test. Comparisons among multiple groups were performed using one-way or two-way ANOVA followed by Tukey's post hoc test. Specific statistical methods for quantitative experiments were indicated in figure legends. *P*-values ≤ 0.05 were considered statistically significant.

## Results

### *MC1R* disruption exacerbates synucleinopathies in the nigrostriatal pathway in αSyn AAV-injected mice

Our previous study demonstrates that the nigrostriatal dopaminergic pathway is compromised under basal conditions in *MC1R*^e/e^ mice carrying a loss-of-function gene mutation and exhibiting blond-red fur [20]. To further investigate how MC1R defends the dopaminergic pathway against αSyn, we injected human WT αSyn AAV into the SN of 5–6-month-old *MC1R*^e/e^ mice and their WT littermates [24]. Expression of human αSyn was detected in the ipsilateral SN 4 weeks post-injection (Fig. S1A). Quantification of fluorescence double-labeling for human αSyn and TH, a marker of dopaminergic neurons, showed 70% and 67% transduction efficiencies for SN dopaminergic neurons in WT and *MC1R*^e/e^ mice, respectively (Fig. S1A and B). By contrast, only few astrocytes in the SN were transduced on the ipsilateral side in WT and *MC1R*^e/e^ mice as shown by double-labeling for human αSyn and GFAP, a marker of glial cells (Fig. S1C). Similarly, there was almost no colocation of αSyn and iba1, a marker of microglia, 4 weeks post-injection (Fig. S1D). These results are consistent with the reported neural tropism of AAV8 [42, 43].

**Fig. 1:**
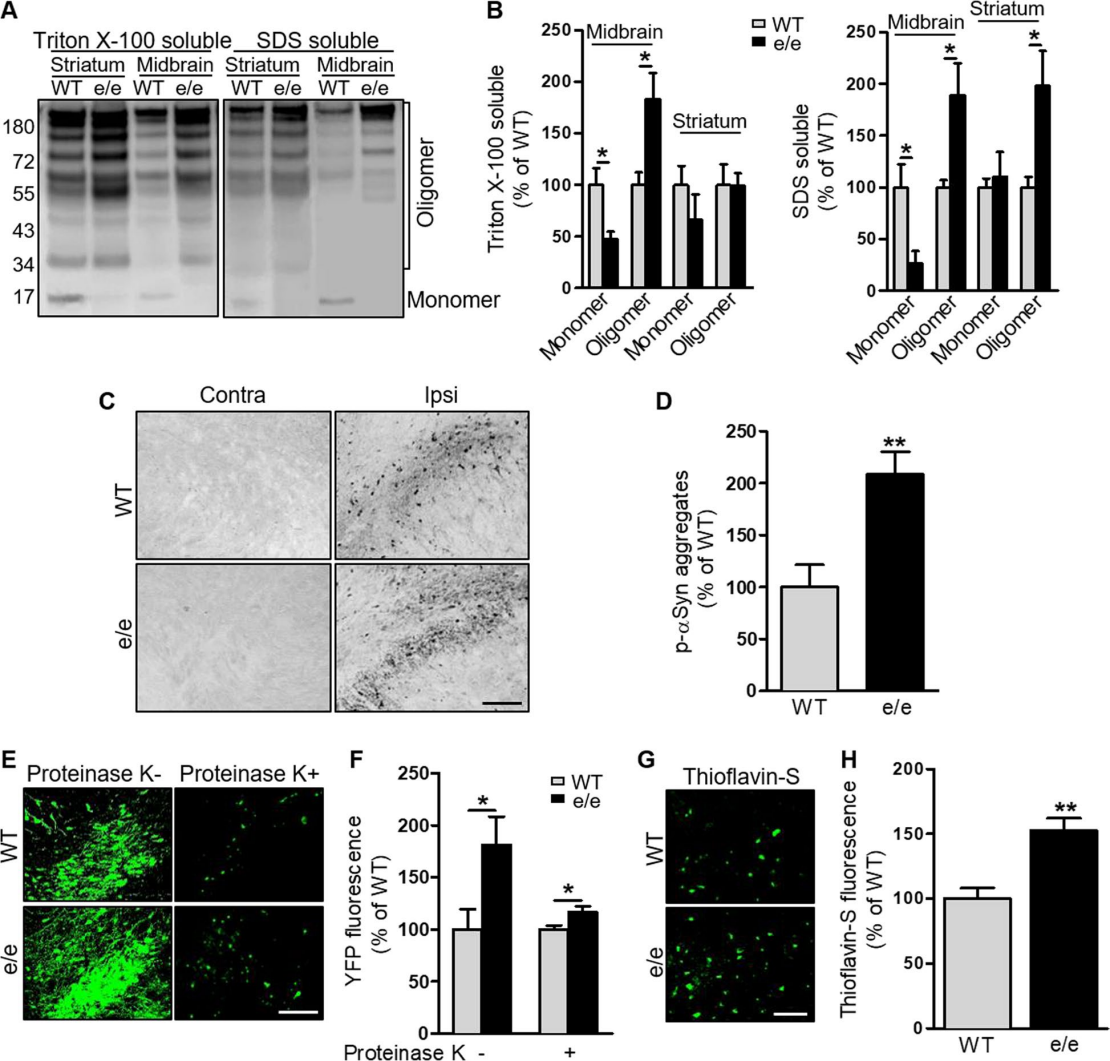
MC1R disruption exacerbates synucleinopathies in the nigrostriatal pathway in an αSyn AAV mouse model. *MC1R*^e/e^ and WT mice were injected unilaterally with human WT αSyn AAV into the SN and sacrificed 8 weeks later: **A** Immunoblot of human αSyn species in Triton X-100-soluble and -insoluble SDS-soluble fractions in ipsilateral ventral midbrain and striatum and **B** quantification of αSyn monomers and oligomers. *n* = 3 mice/group. Measurements were normalized by dividing values by the mean of WT and multiplying by 100. One-way ANOVA followed by Tukey's post hoc test. **C** p-αSyn staining and **D** quantification of p-αSyn aggregates in the ipsilateral SN. *n* = 3 mice/group. Measurements were normalized by dividing values by the mean of WT and multiplying by 100. Two-tail Student’s *t*-test. Scale bar, 50 µm. *MC1R*^e/e^ and WT mice were injected unilaterally with BiFC human WT αSyn AAV into the SN and sacrificed 8 weeks later: **E** VenusYFP fluorescence before and after proteinase K treatment and **F** quantification of venusYFP fluorescence density in the ipsilateral SN. *n* = 4 mice/group. Measurements were normalized by dividing values by the mean of WT and multiplying by 100. One-way ANOVA followed by Tukey's post hoc test. Scale bar, 50 µm. **G** Thioflavin-S staining and **H** quantification of thioflavin-S fluorescence density in the ipsilateral SN. *n* = 4 mice/group. Measurements were normalized by dividing values by the mean of WT and multiplying by 100. Two-tail Student’s *t*-test. Scale bar, 50 µm. *P < 0.05, **P < 0.01

High molecular weight αSyn species were detected by sequential tissue extraction followed by Western blotting using an antibody against human αSyn 8 weeks after AAV injection. Consistent with our previous results, αSyn overexpression in WT mice was associated with αSyn aggregation in the ventral midbrain and striatum on the injection side [24]. Higher molecular weight αSyn species were significantly more abundant in both Triton-soluble and -insoluble SDS-soluble fractions from the injection side in *MC1R*^e/e^ mice compared with WT mice (Fig. 1A and B,  Fig. S1E). Profound p-αSyn, which is associated with aging and synucleinopathies [44, 45], was detected by immunohistochemistry in the ipsilateral SN in *MC1R*^e/e^ mice. Quantification showed increased p-αSyn accumulation in the SN of *MC1R*^e/e^ mice compared with WT mice (Fig. 1C and D).

A different set of *MC1R*^e/e^ and WT mice received injections of human WT αSyn AAV fused to the N-terminus and C-terminus halves of venus YFP into the SN, and αSyn aggregation was visualized by conjugated fluorescence. Our previous study demonstrated that the dopaminergic phenotype induced by this BiFC αSyn is comparable to that in non-BiFC αSyn mouse models [24]. Profound reconstituted YFP fluorescence was observed 8 weeks after AAV injection (Fig. 1E and F). Fluorescence intensity was higher in the ipsilateral SN of *MC1R*^e/e^ mice than in that of WT mice (Fig. 1E and F). Proteinase K digestion revealed the soluble nature of the vast majority of αSyn oligomers. However, *MC1R*^e/e^ mice had significantly higher levels of proteinase K-resistant αSyn oligomers than WT mice (Fig. 1E and F). Thioflavin-S dyeing provided further evidence of more amyloid fibrils in the ipsilateral SN in *MC1R*^e/e^ mice compared with WT mice (Fig. 1G and H). Collectively, these results show exacerbated synucleinopathies in the nigrostriatal pathway resulting from *MC1R* loss of function in a αSyn AAV mouse model. No human αSyn transduction or pathology on the contralateral, non-injected side was observed in either *MC1R*^e/e^ or WT mice (Fig. S1A).

### *MC1R* disruption amplifies αSyn overexpression-induced microglia activation and alters Nrf2 response in the nigrostriatal pathway

**Fig. 2:**
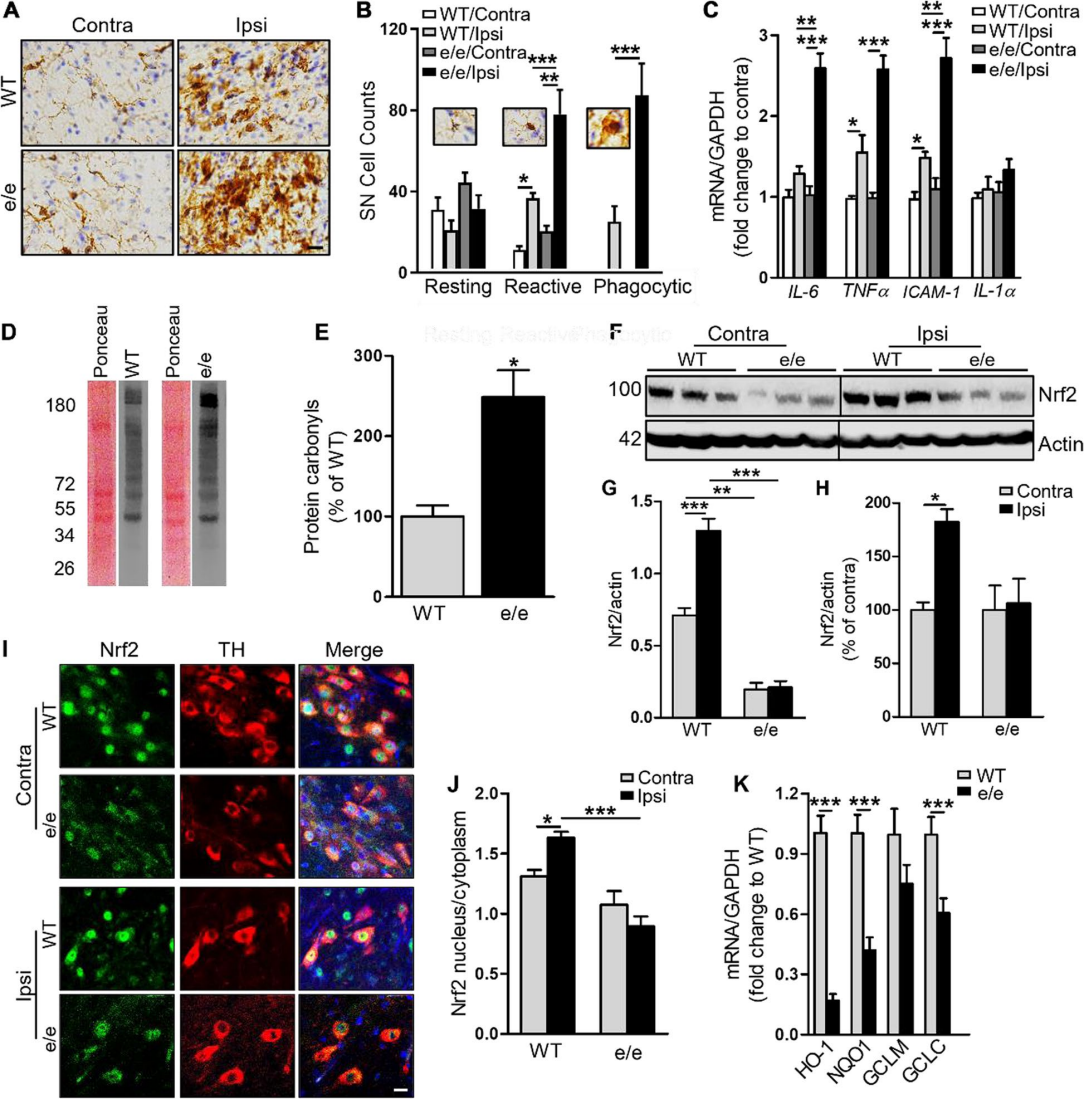
*MC1R* disruption amplifies microglia activation and alters Nrf2 response to αSyn overexpression in the nigrostriatal pathway. *MC1R*^e/e^ and WT mice were injected unilaterally with human WT αSyn AAV into the SN and sacrificed 8 weeks later: **A** Iba1 staining and **B** morphological classification and quantification of iba1-positive cells in the SN. *n* = 4 mice/group. Two-way ANOVA followed by Tukey’s post hoc test. Scale bar, 30 µm. **C** IL-1a, IL-6, TNFα, and ICAM1 mRNA levels in ventral midbrain. Measurements were normalized by dividing values by the mean of the WT contralateral side. One-way ANOVA followed by Tukey’s post hoc test. *n* = 5 mice/group. **D** Representative oxyblots for protein carbonyls and the corresponding Ponceau S staining in the ipsilateral ventral midbrain and **E** quantification of band density. Measurements were normalized by dividing values by the mean of WT control and multiplying by 100. Two-tail Student’s *t*-test. *n* = 3 mice/group. **F** Immunoblot for Nrf2 using ventral midbrain tissue and **G** quantification of Nrf2 band density in original values or **H** normalized to contralateral side by dividing values by the mean of the contralateral side and multiplying by 100. One-way ANOVA followed by Tukey’s post hoc test. *n* = 3 mice/group.**I** SN sections double-stained for Nrf2 and TH and **J** quantification of nuclear and cytoplasmic Nrf2. One-way ANOVA followed by Tukey’s post hoc test. *n* = 3 mice/group. Scale bar, 20 µm. **K** mRNA levels of Nrf2 target genes HO-1, NQO1, GCLM, and GCLC in the ipsilateral ventral midbrain. Measurements were normalized by dividing values by the mean of WT control. One-way ANOVA followed by Tukey’s post hoc test. *n* = 5 mice/group. *P < 0.05, **P < 0.01, ***P < 0.001

Oxidative stress and activation of the immune system, which involve MC1R signaling [10, 12], contribute to neurodegeneration in PD. To determine whether *MC1R* disruption is associated with altered inflammation and oxidative stress in response to αSyn AAV injection, we assessed microglial cells by immunostaining for iba1 8 weeks after injection. Consistent with our previous report [24], αSyn overexpression increased iba1 immunoreactivity on the ipsilateral side compared with the contralateral side. Also, an overall enhanced iba1 signal was observed in *MC1R*^e/e^ mice compared with WT mice (Fig. 2A and B). Iba1-positive cells were classified based on their morphology, and numbers of cell subtypes were counted. Numbers of reactive and phagocytic microglia on the injection side were dramatically increased in *MC1R*^e/e^ mice. There were also trends toward increased numbers of resting and reactive microglia on the contralateral, non-injected side in *MC1R*^e/e^ mice compared with WT mice, indicating possible basal microgliosis due to *MC1R* disruption. Venus or Vector did not induce microgliosis either in WT or *MC1R*^e/e^ mice (Fig. S2A and C).

Integrated optical density of GFAP staining in the SN revealed increased GFAP immunoreactivity in WT mice (Fig. S3A), consistent with the previously reported astrogliosis following αSyn overexpression by our group and others [24, 45]. A similar increase in GFAP immunoreactivity was identified in *MC1R*^e/e^ mice, both in absolute value and after normalization for increased GFAP on the contralateral side (Fig. S3B and C). There were no significant differences on the ipsilateral side between genotypes (Fig. S3C). Venus or Vector did not induce astrogliosis or dopaminergic deficits either in WT or *MC1R*^e/e^ mice (Fig. S2A and B, Fig. S2D, E, F and G).

mRNA levels of the pro-inflammatory cytokines interleukin (IL)-6, tumor necrosis factor (TNF)α, intercellular adhesion molecule 1 (ICAM1), and IL-1α in the ventral midbrain were analyzed 8 weeks after AAV injection using qPCR. αSyn overexpression increased levels of IL-6, TNFα, and ICAM-1 on the ipsilateral side compared with the contralateral side in both *MC1R*^e/e^ and WT mice (not significant for IL-1α). Compared with WT mice, *MC1R*^e/e^ mice exhibited significantly higher levels of IL-6, TNFα, and ICAM1 in the ipsilateral midbrain (Fig. 2C). These results indicate that *MC1R* dysfunction alters the immune response to αSyn.

We previously found increased oxidative protein and DNA damage in the *MC1R*^e/e^ mouse brain under basal conditions [20]. Here, we measured protein carbonylation, a general marker of oxidative stress, by Oxyblot in the ipsilateral ventral midbrain of *MC1R*^e/e^ and WT mice injected with αSyn AAV and found more protein carbonyls in *MC1R*^e/e^ mice than in WT mice 8 weeks after injection (Fig. 2D and E; Fig. S3D).

We next assessed transcription factor Nrf2 signaling, a key regulator of antioxidant and anti-inflammatory pathways through its nuclear translocation and activation and subsequent transcriptional activation of target genes [46]. Western blot showed significantly reduced Nrf2 in the ipsilateral ventral midbrain of *MC1R*^e/e^ mice 8 weeks after αSyn AAV injection as compared with WT mice (Fig. 2F and G). Contralateral Nrf2 was also significantly decreased in *MC1R*^e/e^ mice compared with WT mice. Given that no αSyn pathologies were observed on the contralateral side, this difference may reflect compromised Nrf2 signaling due to disrupted MC1R under basal conditions without additional insult. After normalizing for the “basal” difference between genotypes by expressing Nrf2 as a percentage of the contralateral value, αSyn overexpression significantly increased Nrf2 levels on the ipsilateral side in WT mice but not in *MC1R*^e/e^ mice (Fig. 2H).

The localization of Nrf2 in dopaminergic neurons in the SN was assessed by fluorescence double-labeling for Nrf2 and TH. Enhanced nuclear localization and accumulation of Nrf2 in dopaminergic neurons was evident in WT mice 8 weeks after αSyn AAV injection (Fig. 2I and J). *MC1R*^e/e^ mice, however, displayed a lower ratio of nuclear-to-cytoplasmic Nrf2 in TH-positive neurons following αSyn overexpression compared with WT mice. There was no significant change in the ratio between ipsilateral and contralateral sides in *MC1R*^e/e^ mice (Fig. 2J). As expected, the mostly inactive Nrf2 was accompanied by markedly lower mRNA levels of the Nrf2 target genes heme oxygenase 1 (HO1), NAD(P)H quinone dehydrogenase (NQO1), and glutamate-cysteine ligase subunit C (GCLC) in the ipsilateral ventral midbrain in *MC1R*^e/e^ mice as assessed by qPCR (Fig. 2K). Taken together, these results demonstrate the absence of Nrf2 activation following αSyn overexpression in terms of both protein induction and nuclear translocation as well as accompanying oxidative damage and an inflammatory response in *MC1R*^e/e^ mice.

Astrocytic Nrf2 signaling is considered critical in aging and neurodegeneration [4, 47]. However, we did not observe appreciable alteration in Nrf2 immunoreactivity overlapping with GFAP in the SN between the contralateral and ipsilateral sides in either WT or *MC1R*^e/e^ mice (Fig. S3E).

### *MC1R* disruption exacerbates αSyn-induced dopaminergic neurotoxicity, which is reversed by human *MC1R* transgene

**Fig. 3:**
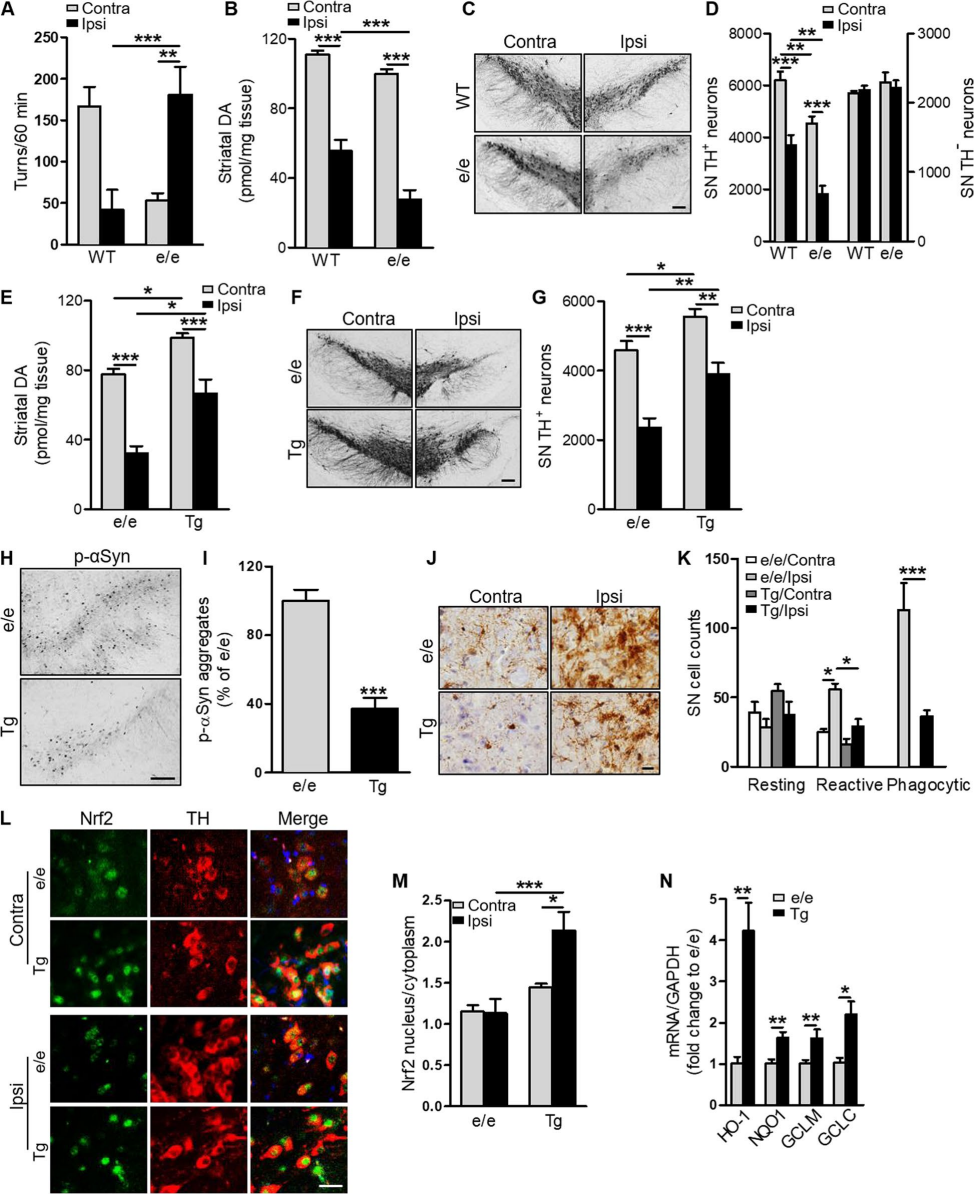
αSyn-induced dopaminergic neurotoxicity is exacerbated by *MC1R* disruption and reversed by human *MC1R* transgene. *MC1R*^e/e^ and WT mice were injected unilaterally with human WT αSyn AAV into the SN. **A** Contralateral and ipsilateral turns induced by amphetamine 12 weeks post-AAV injection. Two-way ANOVA followed by Tukey’s post hoc test. *n* = 12 mice/group. **B** Striatal dopamine content 16 weeks post-AAV injection. Two-way ANOVA followed by Tukey’s post hoc test. *n* = 12 mice/group. **C** TH staining and **D** stereological quantification of TH-positive and -negative cells in the SN 16 weeks post-AAV injection. Two-way ANOVA followed by Tukey’s post hoc test. *n* = 12 mice/group. Scale bar, 100 µm. *MC1R*^e/e^Tg and *MC1R*^e/e^ mice were injected unilaterally with human WT αSyn AAV into the SN. **E** Striatal dopamine content 16 weeks post-AAV injection. Two-way ANOVA followed by Tukey’s post hoc test. *n* = 6–7 mice/group. **F** TH staining and **G** stereological quantification of TH-positive and negative cells in the SN 16 weeks post-AAV injection. Two-way ANOVA followed by Tukey’s post hoc test. *n* = 6–7 mice/group. Scale bar, 100 µm. **H** p-αSyn staining and **I** quantification of p-αSyn aggregates in the ipsilateral SNpc 12 weeks post-AAV injection. Measurements were normalized by dividing values by the mean of the *MC1R*^e/e^ mice and multiplying by 100. Two-tail Student’s *t* test. *n* = 4 mice/group. Scale bar, 50 µm. **J** Iba1 staining and morphological classification and **K** quantification of iba1-positive cells in the SNpc 12 weeks post-AAV injection. Two-way ANOVA followed by Tukey’s post hoc test. *n* = 4 mice/group. Scale bar, 30 µm. **L** Nrf2 and TH double-labeling and (M) quantification of nuclear and cytoplasmic Nrf2 in the SNpc 12 weeks post-AAV injection. Two-way ANOVA followed by Tukey’s post hoc test. *n* = 4 mice/group. Scale bar, 20 µm. **N** mRNA levels of Nrf2 target genes HO-1, NQO1, GCLC, and GCLM in the ipsilateral ventral midbrain 12 weeks post-AAV injection. Measurements were normalized by dividing values by the mean of the *MC1R*^e/e^ mice. One-way ANOVA followed by Tukey’s post hoc test. *n* = 5 mice/group. *P < 0.05, **P < 0.01, ***P < 0.001

We next assessed dopaminergic neurotoxicity due to αSyn overexpression in *MC1R*^e/e^ and WT mice. Six-month-old mice were injected unilaterally with human WT αSyn AAV in the SN. Rotational behavior induced by the dopamine-releasing agent amphetamine was analyzed 12 weeks following AAV injection. *MC1R*^e/e^ mice displayed a tendency to rotate toward the ipsilateral side after AAV injection, whereas WT mice tended to rotate toward the contralateral side (Fig. 3A), as previously reported [24]. There was a significant difference in the number of ipsilateral turns between genotypes. When we measured dopamine content in the striatum by HPLC at 16 weeks, we found that αSyn overexpression induced significantly greater DA depletion on the ipsilateral side in *MC1R*^e/e^ mice than in WT mice either in absolute value (Fig. 3B) or percentage of the contralateral side (Fig. S3F). When we analyzed dopaminergic neuron survival by stereological counting of TH-positive cells in the SN, we found a more substantial and significant loss of dopaminergic neurons in *MC1R*^e/e^ mice compared with WT mice either in absolute value (Fig. 3C and D) or percentage of the contralateral side (Fig. S3G). Mild-to-moderate reductions in striatal dopamine and nigral dopaminergic neuron counts on the contralateral side in *MC1R*^e/e^ mice are consistent with our previously reported basal dopaminergic defects [20] assuming that the contralateral side is similar to the intact condition, as no contralateral αSyn transduction was detected (Fig. S1A). Representative images of ventral midbrain TH staining show extensive loss of TH‐positive cells in a *MC1R*^e/e^ mouse (Fig. 3C). Nissl counterstaining and stereological counting of TH‐negative neurons in the SNpc revealed no difference between *MC1R*^e/e^ and WT mice, suggesting that the deleterious effects of *MC1R* disruption and its interaction with αSyn are dopaminergic-specific, at least in the ventral midbrain (Fig. 3D).

To investigate whether *MC1R* disruption is causative of higher αSyn susceptibility and to explore the potential of human *MC1R* to reverse the *MC1R* loss-of-function phenotypes, we employed Tg mice expressing human *MC1R* using the human promoter to simulate physiological expression in humans [23]. The transgene reverses the *MC1R* deficiency yellow/red pigmentation phenotype to give Tg mice in an e/e background (*MC1R*^e/e^Tg) a WT-like dark coat. We first confirmed transgene expression in *MC1R*^e/e^Tg mice using human MC1R specific antibody. Robust human MC1R was detected in the SN, mostly in the cytoplasm as well as on the cell surface. This transgene expression pattern was similar to that of the endogenous mouse receptor stained using non-human specific antibody in WT adult C57BL/6 J mice (Fig. S4A) and is consistent with our previous report [20]. Double-labeling for TH and MC1R showed the presence of Tg MC1R in dopaminergic neurons in the SN (Fig. S4A). Double-labeling for GFAP and MC1R showed that most GFAP-positive cells were in the SN pars reticulata, where only a few cells were MC1R-positive. Few GFAP-positive astrocytes co-expressed Tg human MC1R (Fig. S4B), also similar to the endogenous expression pattern of MC1R in WT C57BL/6 J mice (Fig. S4B). Similarly, there appeared to be only negligible overlap between MC1R and iba1 in the SN in either *MC1R*^e/e^Tg or WT mice (Fig. S4C).

We next unilaterally injected 5–6-month-old *MC1R*^e/e^Tg mice and their *MC1R*^e/e^ littermates with αSyn AAV. Similar αSyn transduction efficiency was confirmed on the injection side in *MC1R*^e/e^Tg and *MC1R*^e/e^ mice by double-staining for human αSyn and TH (Fig. S4D and E) 4 weeks after injection. Double-labeling for human αSyn and GFAP showed that few astrocytes in the SN were transduced in either *MC1R*^e/e^Tg or *MC1R*^e/e^ mice (Fig. S4F).

Dopaminergic phenotypes were assessed 16 weeks after αSyn AAV injection. *MC1R*^e/e^Tg mice showed 33% dopamine depletion on the ipsilateral side as compared with 59% dopamine depletion in *MC1R*^e/e^ mice after normalizing to the contralateral sides within each genotype to eliminate the likely basal difference between genotypes, similar to the comparison between WT and *MC1R*^e/e^ mice (Fig. 3E and Fig. S4G). A higher percentage of surviving SN dopaminergic neurons after AAV injection was also observed in *MC1R*^e/e^Tg mice (70%) as compared with *MC1R*^e/e^ mice (51%) (Fig. 3F and G, Fig. S4H). Differences in contralateral striatal dopamine level and dopaminergic neuron counts between genotypes likely reflect the restoration of previously reported deficits in *MC1R*^e/e^ mice under basal conditions (Fig. 3E, F and G) [20].

To confirm human *MC1R* rescue of αSyn neurotoxicity in the e/e background, we assessed αSyn pathology, neuroinflammation, and Nrf2 in the SN in *MC1R*^e/e^Tg and *MC1R*^e/e^ mice. Twelve weeks after αSyn AAV injection, *MC1R*^e/e^Tg mice showed significantly less p-αSyn aggregates on the ipsilateral side compared with *MC1R*^e/e^ mice (Fig. 3H and I). No p-αSyn staining was observed on the contralateral side in either *MC1R*^e/e^Tg or *MC1R*^e/e^ mice. Iba1 immunoreactivity was increased overall on the ipsilateral side compared with the contralateral side in both *MC1R*^e/e^Tg and *MC1R*^e/e^ mice 12 weeks after AAV injection. Quantification of morphology-based cell subtypes revealed a significant reduction in the numbers of reactive and phagocytic microglia in the injected SN in *MC1R*^e/e^Tg mice compared with *MC1R*^e/e^ mice (Fig. 3J and K).

Double-immunostaining for Nrf2 and TH showed a significantly higher nuclear-to-cytoplasmic Nrf2 ratio on the ipsilateral side in *MC1R*^e/e^Tg mice compared with *MC1R*^e/e^ mice 12 weeks after AAV injection (Fig. 3L and M). qPCR analysis showed induction of Nrf2 target genes *HO-1, NQO1, GCLM,* and *GCLC* at the mRNA level in the ipsilateral ventral midbrain in *MC1R*^e/e^Tg mice compared with *MC1R*^e/e^ mice (Fig. 3N). Nrf2 signal within GFAP-positive cells in the SN did not seem to be prominent on either side in *MC1R*^e/e^ and did not appear to be altered in *MC1R*^e/e^Tg mice (Fig. S4I).

### Pharmacological MC1R activation is neuroprotective against αSyn-induced dopaminergic neurotoxicity

**Fig. 4:**
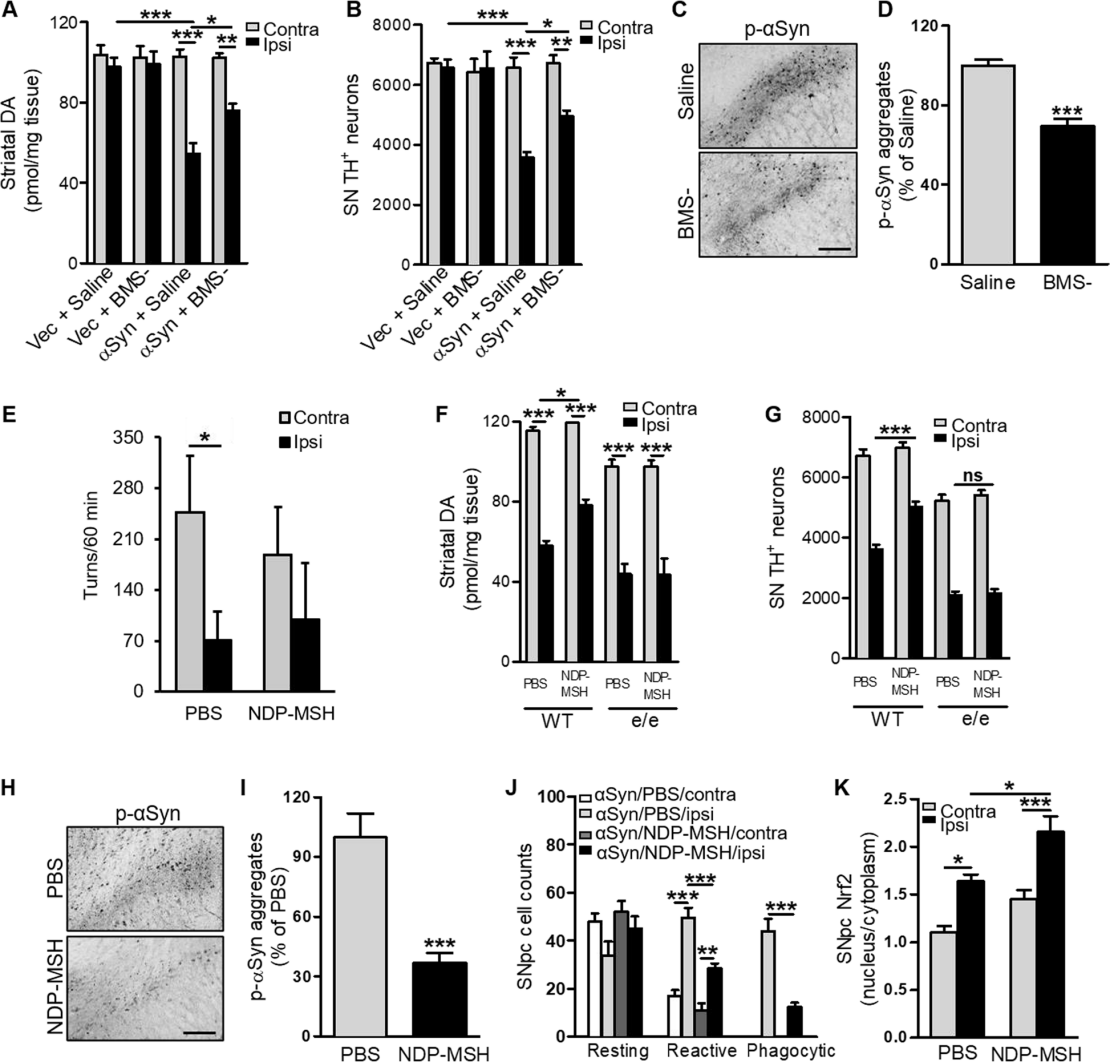
MC1R agonists protect against αSyn-induced dopaminergic neurotoxicity. C57Bl/6 J mice were injected with αSyn or empty vector (Vec) AAV, treated subcutaneously with 20 mg/kg BMS-470539 (BMS-) or saline for 4 weeks, and sacrificed 16 weeks post-AAV injection. **A** Striatal dopamine content. Two-way ANOVA followed by Tukey’s post hoc test. *n* = 6–8 mice/group. **B** Stereological quantification of TH-positive cells in the SN. Two-way ANOVA followed by Tukey’s post hoc test. *n* = 6–8 mice/group. **C** p-αSyn staining and **D** quantification of p-αSyn aggregates in the ipsilateral SN. Measurements were normalized by dividing values by the mean of the vehicle-treated group and multiplying by 100. Two-tailed Student’s *t* test. *n* = 4 mice/group. Scale bar, 50 µm. WT and *MC1R*^e/e^ mice were injected unilaterally with 3 nmol NDP-MSH into the striatum and then with αSyn AAV into the SN. Mice were sacrificed 16 weeks post-AAV injection. **E** Contralateral and ipsilateral turns induced by amphetamine 12 weeks post-AAV injection. Two-way ANOVA followed by Tukey’s post hoc test. *n* = 7 mice/group. **F** Striatal dopamine content. Two-way ANOVA followed by Tukey’s post hoc test. *n* = 7 mice/group. **G** Stereological quantification of TH-positive cells in the SN. Two-way ANOVA followed by Tukey’s post hoc test. *n* = 7 mice/group. **H** p-αSyn staining and **I** quantification of p-αSyn aggregates in the ipsilateral SN. Measurements were normalized by dividing values by the mean of the vehicle-treated group and multiplying by 100. Two-tail Student’s *t* test. *n* = 4 mice/group. Scale bar, 50 µm. **J** Morphological classification and quantification of iba1-positive cells in the SN in WT mice. Two-way ANOVA followed by Tukey’s post hoc test. *n* = 4 mice/group. **K** Nuclear and cytoplasmic Nrf2 ratio in the SN in WT mice. Two-way ANOVA followed by Tukey’s post hoc test. *n* = 4 mice/group. *P < 0.05, **P < 0.01, ***P < 0.001

This proof-of-concept genetic rescue provides rationale for MC1R as a therapeutic target for PD. We previously reported a dopaminergic neuroprotective effect of systemically administered MC1R agonist BMS-470539 in a MPTP mouse model of PD [20]. BMS-470539 is MC1R-selective and has ~ 10% brain penetrance. C57Bl/6 J mice were injected with αSyn AAV and treated daily with BMS-470539 for 4 weeks at a dose of 20 mg/kg subcutaneously. αSyn reduced striatal DA on the ipsilateral side in saline-treated mice 16 weeks after AAV injection, whereas BMS-470539 treatment significantly attenuated the αSyn-induced DA deficit (Fig. 4A). Stereological counting of TH-positive cells revealed a significantly higher number of surviving SN dopaminergic neurons in BMS-470539-treated mice compared with saline-treated mice (Fig. 4B). Reduced p-αSyn aggregates were observed on the ipsilateral side in BMS-470539-treated mice compared with saline-treated mice (Fig. 4C and D). Consistent with our previous report [24] injection of empty vector AAV did not induce significant depletion of striatal dopamine or loss of SN dopaminergic neurons. Moreover, BMS-470539 treatment did not alter striatal dopamine content or SN dopaminergic neuron counts in vector-injected mice (Fig. 4A and B).

We next tested another MC1R agonist, NDP-MSH, in our αSyn AAV mouse model. In a pharmacokinetic study with C57Bl/6 J mice, no NDP-MSH was detected in the brain at any time point from 5 to 120 min after 1 mg/kg intraperitoneal injection (*n* = 3/time point). NDP-MSH was administered intracranially into the left striatum at 3 nmol before αSyn AAV injection in WT C57Bl/6 J mice. Amphetamine-induced rotational behavior was analyzed 12 weeks following AAV injection. Consistent with findings in Fig. 3A and previous report [24], control vehicle-treated mice tended to rotate toward the contralateral side. Although there was no statistical difference in either ipsilateral turns or contralateral turns between NDP-MSH and vehicle groups, NDP-MSH treated mice did not show significant difference in the numbers of ipsilateral and contralateral turns as the control mice did (Fig. 4E). NDP-MSH treatment mildly but significantly increased striatal dopamine and SN dopaminergic neuron count on the ipsilateral side 16 weeks after AAV injection compared with PBS-treated mice (Fig. 4F and G). When we assessed p-αSyn and iba1 in the ipsilateral SN by immunohistochemistry, we found dramatically reduced p-αSyn in NDP-MSH-treated mice compared with PBS-treated mice (Fig. 4H and I), and this reduction in p-αSyn on the ipsilateral side was accompanied by only modest microglia activation as reflected by a decreased number of reactive and phagocytic microglia (Fig. 4K). The nuclear**-**to**-**cytoplasmic Nrf2 ratio on the ipsilateral side in NDP-MSH-treated mice (2.2) was significantly higher than that in PBS-treated mice (1.6) (Fig. 4K). The same treatment regimen in *MC1R*^e/e^ mice did not result in differences in striatal dopamine or SN TH-positive cell counts between NDP-MSH- and PBS-treated mice (Fig. 4F and G), indicating that functioning MC1R is required for mediating the protective effects of NDP-MSH despite the broad affinity of NDP-MSH for other melanocortin receptors.

### MC1R activation alleviates αSyn oligomerization and neurotoxicity by activating Nrf2 in vitro

**Fig. 5:**
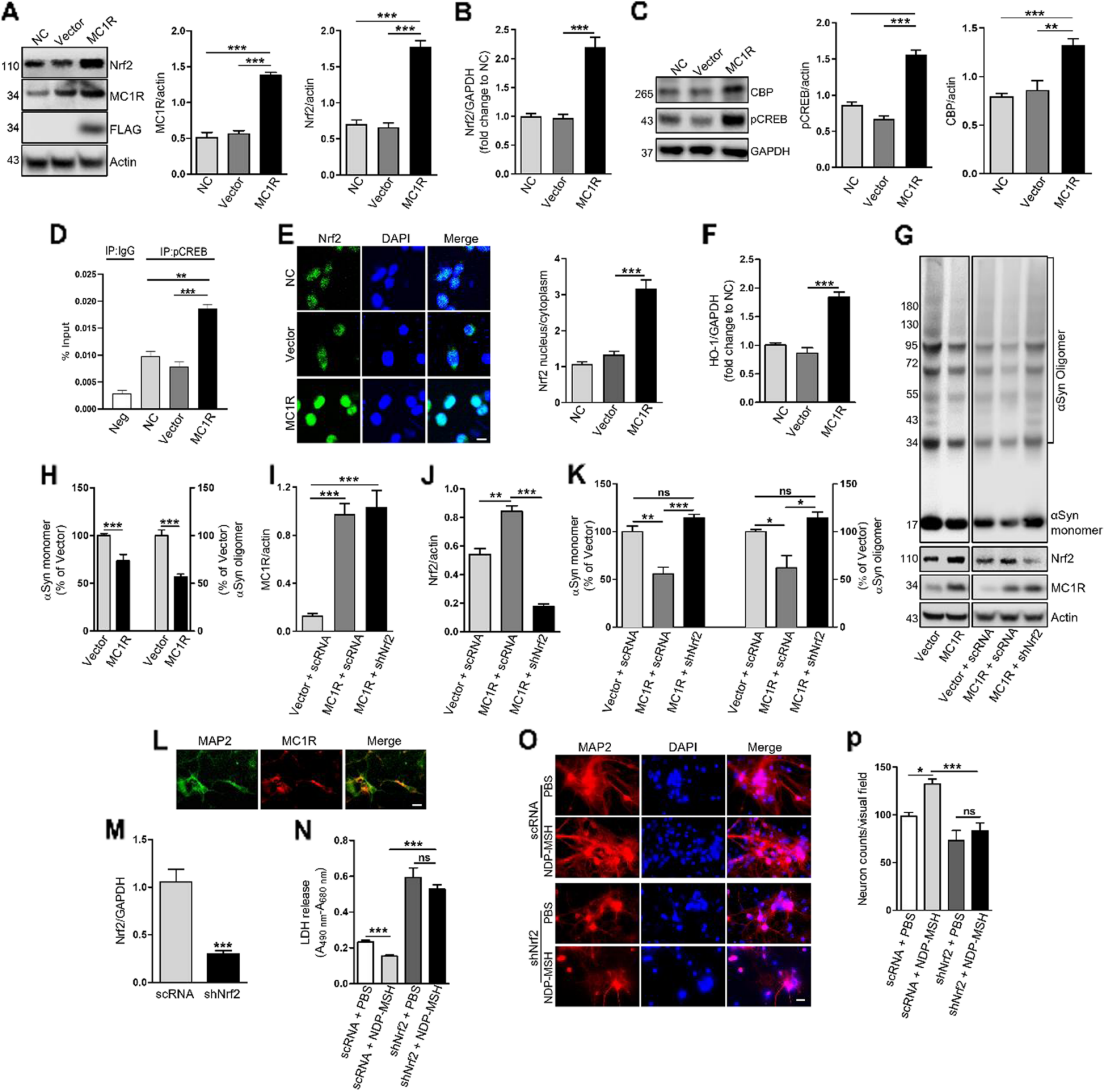
MC1R activation alleviates αSyn oligomerization and neurotoxicity by activating Nrf2 in vitro. HEK293T cells were co-transfected with αSyn and MC1R or vector (GPRC5A-Tango). Non-transfected cells served as controls (NC): **A** Immunoblot and quantification of MC1R and Nrf2. One-way ANOVA followed by Tukey's post hoc test. **B** Nrf2 mRNA levels. One-way ANOVA followed by Tukey's post hoc test. **C** Immunoblot and quantification of pCREB and CBP. One-way ANOVA followed by Tukey's post hoc test. **D** ChIP-qPCR analysis of pCREB binding in the Nrf2 promoter. Chromatins were immunoprecipitated using pCREB or IgG as negative control (Neg). Values were calculated by subtracting the cycle threshold (Ct) values for immunoprecipitated DNA from adjusted Ct of input DNA to get delta Ct, followed by raising 2 to the power of the delta Ct and multiplying by 100. One-way ANOVA followed by Tukey's post hoc test. **E** Nrf2 staining and quantification of nuclear and cytoplasmic Nrf2. Nuclei were stained with DAPI. One-way ANOVA followed by Tukey's post hoc test. Scale bar, 10 µm. **F** Nrf2 target gene HO-1 mRNA levels. One-way ANOVA followed by Tukey's post hoc test. **G** and **H** Immunoblot and quantification of αSyn species. Measurements were normalized by dividing values by the mean of vector control and multiplying by 100. One-way ANOVA followed by Tukey's post hoc test. HEK293T cells were transfected with αSyn, MC1R or vector control, and shNrf2 RNA or scRNA control: **G** Immunoblot and **I** and **J** quantification of MC1R and Nrf2. One-way ANOVA followed by Tukey's post hoc test. **G** Immunoblot and **K** quantification of αSyn species. Measurements were normalized by dividing values by the mean of vector control and multiplying by 100. One-way ANOVA followed by Tukey's post hoc test. Primary cortical neurons were prepared from embryonic day 16–17 WT mice: **L** MAP2 and MC1R staining at DIV5. Scale bar, 10 µm. (M) Nrf2 mRNA levels in primary neurons transduced with αSyn AAV and lentiviral scRNA or shNrf2 RNA. Two-tail Student’s t test. **N** LDH release in primary neurons transduced with αSyn AAV and lentiviral scRNA or shNrf2 RNA and treated with PBS or NDP-MSH. Two-way ANOVA followed by Tukey’s post hoc test. **O** MAP2 staining and **N** quantification of MAP2-postive cells in primary neurons transduced with αSyn AAV and lentiviral scRNA or shNrf2 RNA. Two-way ANOVA followed by Tukey’s post hoc test. Scale bar, 10 µm. Visual field area = 0.13508 mm^2^. *P < 0.05, **P < 0.01, ***P < 0.001, ns = not statistically significant. *n* = 3 replicates. Experiments were repeated ≥ 3 times

Given that MC1R genetic and pharmacological manipulations alter Nrf2 signaling, we further explored the role of Nrf2 in the protective actions of MC1R against αSyn pathologies in vitro using the human embryonic kidney cell line HEK293T. Transfection of human WT αSyn resulted in expression of human αSyn in both monomer and oligomer forms as demonstrated by Western blotting after crosslinking to stabilize αSyn oligomers (Fig. S5A). Endogenous αSyn in HEK293T cells is limited or undetectable [48]; no αSyn was detected in non-transfected or vector-transfected cells (Fig. S5A). Co-transfection with human MC1R in HEK293T cells overexpressing αSyn led to a significant increase in Nrf2 compared with non-transfected or vector-transfected cells at both the protein and mRNA levels as determined by Western blotting and qPCR (Fig. 5A and B). The human MC1R, similar to the mouse receptor, has been shown to have significant ligand-independent signaling when overexpressing in HEK293 cells [23].

Transcriptional factor CREB, whose phosphorylation at Ser133 recruits CBP and enhances target gene activation [49], is known to respond to MC1R activation [50]. We assessed pCREB (Ser133) and CBP by Western blotting and found a significant increase in both pCREB and CBP in αSyn expressing HEK293T cells co-transfected with MC1R compared with non-transfected or vector-transfected cells (Fig. 5C). Previous whole-genome ChIP-chip analyses of CREB binding genes identified CREB binding sites in the *Nrf2* promoter region [51] [http://natural.salk.edu/creb/]. To explore transcriptional activation of Nrf2 by MC1R through CREB in our in vitro system, we performed ChIP assay using pCREB (Ser133) antibody. qPCR analysis of pCREB-immunoprecipitated chromatins using primers for CREB binding site (Fig. S5B) demonstrated significantly higher association of CREB and the *Nrf2* promoter in MC1R transfected cells as compared to non-transfected and vector controls (Fig. 5D). Fluorescence staining showed an increased ratio of nuclear-to-cytoplasmic Nrf2 (Fig. 5E). In addition, qPCR showed increased HO-1 mRNA expression in MC1R and αSyn co-transfected cells (Fig. 5F). These results suggest that Nrf2 is induced and activated by MC1R overexpression in HEK293T cells overexpressing αSyn. Immunoblotting for human αSyn showed significantly reduced αSyn in both its monomer form and higher molecular weight species in cells overexpressing MC1R compared with vector-transfected cells (Fig. 5G and H).

We next employed shNrf2 to knock down Nrf2 in HEK293T cells transfected with αSyn and MC1R or control vector. Transduction of shNrf2 or control scRNA was achieved using lentivirus, and overexpression of MC1R and knock down of Nrf2 were confirmed by Western blotting (Fig. 5G, H, I and J). The reduction in αSyn monomer and oligomers by MC1R overexpression was reversed in cells transduced with shNrf2 as assessed by immunoblotting. Quantification revealed no difference in αSyn monomer or oligomers in vector-transfected cells treated with scRNA (Fig. 5G, K). These results suggest that Nrf2 has an essential role in mediating the influence of MC1R on αSyn in HEK293T cells.

To further confirm Nrf2 as a downstream effector of MC1R neuroprotection, we prepared cortical primary neurons from WT and *MC1R*^e/e^ mouse embryos and performed MAP2 and GFAP immunofluorescence staining. A high purity of neurons in the culture was supported by abundant MAP2-postive cells and rare GFAP-stained cells (Fig. S5C). Endogenous MC1R expression in cortical neurons was detected by immunohistochemistry (Fig. 5L). Previous studies reported that MC1R is expressed in CNS neurons in late gestation [52] and neuron cultures prepared from C57BL/6 J embryos [18]. To characterize αSyn toxicity and the effect of MC1R in primary cortical neurons, cell preparations from WT and *MC1R*^e/e^ mice were transduced with BiFC αSyn AAV or control venus AAV at day in vitro (DIV3) and treated with the MC1R agonist NDP-MSH 2 days later. Transduction of venus AAV or αSyn oligomerization and morphological changes were visualized by fluorescence at DIV9. Similar to our previous report in mice [24], oligomeric BiFC αSyn was associated with beaded and punctate structures in WT neurons (Fig. S5D). Consistently, higher cytotoxicity was indicated by a LDH release assay in BiFC αSyn-transduced cells compared with venus-transduced cells at DIV12. Treatment with NDP-MSH attenuated αSyn cytotoxicity. αSyn oligomerization and the related appearance of distorted and segmented neurites were more evident in *MC1R*^e/e^ neurons (Fig. S5D). NDP-MSH treatment of *MC1R*^e/e^ neurons did not alter αSyn cytotoxicity, suggesting that the protective effect of NDP-MSH against αSyn depends on functioning MC1R (Fig. S5E).

We next knocked down Nrf2 in WT primary neurons transfected with αSyn using lentiviral shNrf2. The knock-down efficiency of shNrf2 was confirmed by qPCR (Fig. 5M). Cells were also treated with NDP-MSH for 7 days, which significantly decreased LDH cytotoxicity at DIV12. There was no difference in LDH release between NDP-MSH- and vehicle-treated cells transduced with shNrf2, suggesting that shNrf2 blocked the NDP-MSH effect (Fig. 5N). The requirement of Nrf2 in the protective effect of NDP-MSH against αSyn toxicity was further demonstrated by MAP2-positive cell counting using immunohistochemistry (Fig. 5O and P). The number of surviving neurons after αSyn transfection in the shNrf2 + NDP-MSH group was significantly lower than that in the scRNA + NDP-MSH group. There was no significant difference between shNrf2 + NDP-MSH and shNrf2 + PBS groups (Fig. 5O and P). Treatment with shNrf2 significantly increased LDH and tended to decrease the number of MAP2-positive cells as compared with scRNA in cortical primary neurons (Fig. 5N, O and P), consistent with the previously reported role of endogenous Nrf2 in maintaining neuronal cell survival [4, 5, 53].

### MC1R is present in dopaminergic neurons in the human SN and reduced in PD patients

**Fig. 6:**
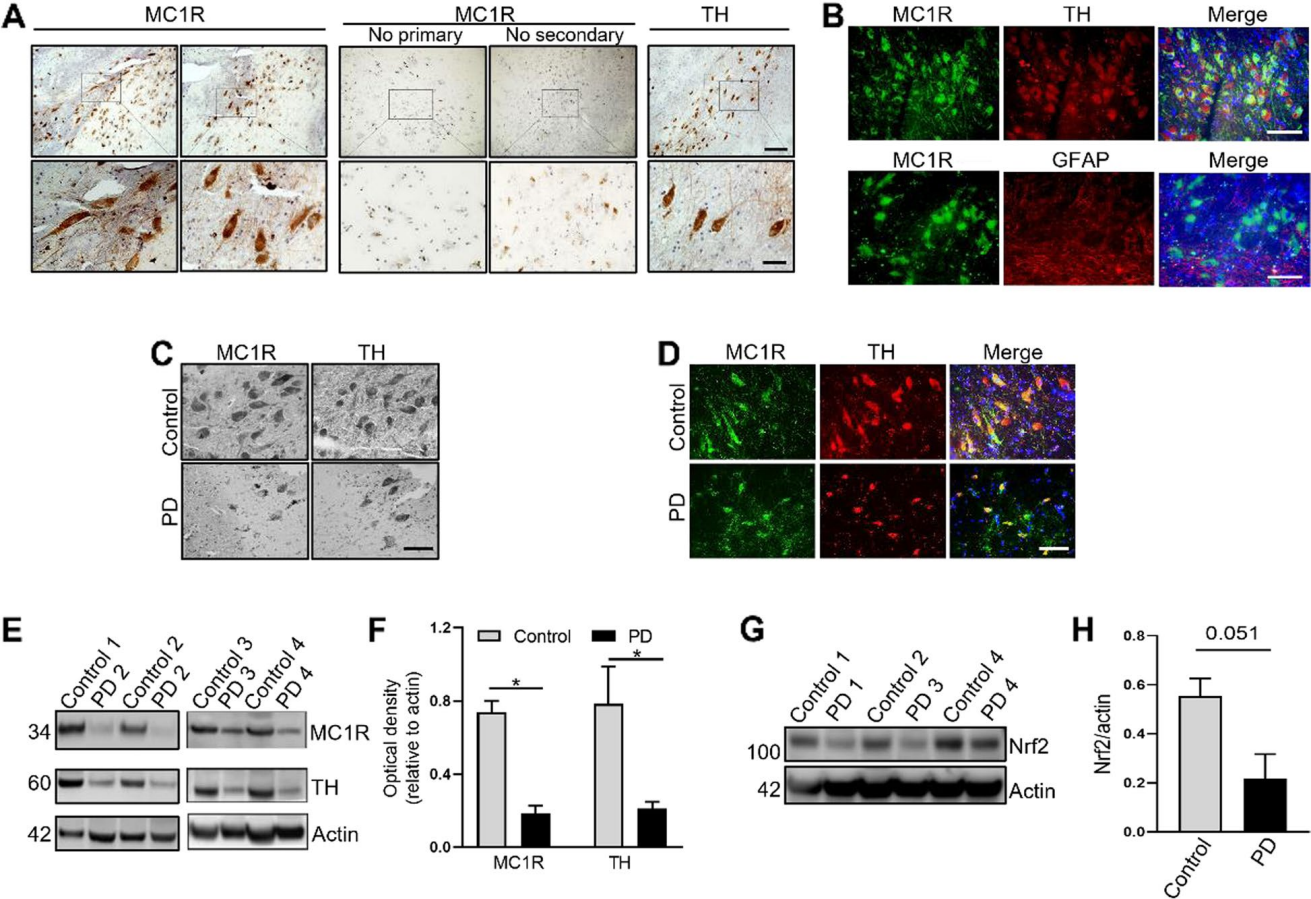
MC1R is present in dopaminergic neurons in humans and is reduced in patients with PD. A Immunohistochemistry for MC1R and TH in the SN of a 60-year-old control individual, PMI 15 h. Control sections were processed in the same manner, except primary or secondary antibodies were omitted. Scale bars, top 100 µm, bottom 25 µm. **B** Fluorescence double-staining for MC1R and TH or GFAP in the SN of a 63-year-old control individual, PMI 16 h. Scale bar, 50 µm. **C** Immunohistochemistry for MC1R and TH in the SN of a 91-year-old control individual, PMI 8 h, and an 84-year-old PD patient, PMI 6 h. Scale bar, 50 µm. **D** Fluorescence double-staining of MC1R and TH in the SN of an 87-year-old control individual, PMI 48 h, and a 91-year-old PD patient, PMI 32 h. Scale bar, 50 µm. **E** Immunoblot for MC1R and TH using SN tissue from control individuals and PD patients and **F** quantification of band density, *n* = 4/group; **G** Immunoblot for Nrf2 using SN tissue from control individuals and PD patients and **H** quantification of band density, *n* = 3/group. Actin as a loading control. Two-tail Student’s *t* test. Age (years)/PMI (h): 63/16, 87/48, 91/8, and 60/15 for control 1–4; 62/33, 91/32, 84/6, and 69/17 for PD 1–4. *P < 0.05

We next determined whether MC1R is expressed in the SN in individuals with no neurological conditions and whether it is altered in patients with PD. Immunohistochemistry showed distinct positive staining for MC1R in the SN, where TH-positive dopaminergic neurons were abundant (Fig. 6A). Examination of cellular morphology revealed that MC1R was expressed on the membrane and in the cytoplasm, similar to our previous report in mice [20], and cell projections were also distinctly stained. Omitting primary or secondary antibody yielded no specific staining, with only dark pigment then visible. Fluorescence staining for MC1R, TH, and nuclear DAPI showed that most TH-positive cells stained positive for MC1R, indicating the presence of MC1R in dopaminergic neurons (Fig. 6B). Fluorescence double-staining showed almost no overlap of MC1R and GFAP (Fig. 6B), consistent with our findings in mice (Fig. S4B).

We next examined postmortem SN tissue from patients with PD. Peroxidase immunostaining indicated fewer MC1R-positive cells in the SN from PD patients compared with controls (Fig. 6C). Loss of TH-positive cells was also evident in the SN of PD patients. After fluorescence double-staining, there appeared to be fewer cells in the SNpc stained with both MC1R and TH compared with controls (Fig. 6C and D). Western blotting using SN tissue showed a loss of MC1R in PD patients, which was accompanied by reduced TH and reduce Nrf2 that was marginally non-significant compared with controls (Fig. 6E, F, G and H, Fig. S6A and B). To determine whether MC1R was altered in AAV αSyn mice prior to significant dopaminergic neuron loss, we assessed MC1R expression by Western blotting at 6 weeks following AAV αSyn injection and found no significant difference in MC1R or TH in the ventral midbrain between the contralateral non-injection side and ipsilateral injection side (Fig. S6C and D).

## Discussion

We previously reported protective effects of melanoma-related MC1R in nigrostriatal dopaminergic neuron survival under basal conditions and in toxin models of PD [20]. In the present study, we found exacerbated αSyn pathology following AAV-mediated overexpression of αSyn in the SN of *MC1R*^e/e^ mice carrying a loss-of-function mutation. Increased oligomeric αSyn, especially amyloid-like αSyn fibrils and p-αSyn, indicated pathologic αSyn aggregation in *MC1R*^e/e^ mice. Existing evidence connecting MC1R and αSyn is scarce and indirect. In vitro studies using melanoma cells, which express MC1R, indicates that αSyn is highly expressed and promotes melanoma cell survival [54, 55]. In addition, αSyn is shown to reduce UV-induced melanin synthesis in melanoma cells, suggesting a possible inhibitory effect of αSyn on the melanin pathway that is controlled by MC1R [56].

As a consequence of exacerbated αSyn pathology, *MC1R*^e/e^ mice with SN-targeted overexpression of αSyn displayed exacerbated dopaminergic deficits anatomically and neurochemically. This exacerbation cannot be solely explained by a preexisting dopaminergic dysfunction previously demonstrated in these mice around the same age [20]. The ability of human *MC1R* to rescue the dopamine deficits indicates the specificity of this MC1R effect. The human *MC1R* transgene, under its human physiological promoter, expresses less but more potent MC1R than the mouse gene and restores pigmentation in *MC1R*^e/e^ mice [23]. Although our results suggest that the SN expression pattern of the transgenic human *MC1R* was similar to the endogenous WT mouse *MC1R* and exerted a protective effect, they do not indicate whether the rescue was partial or complete or whether the transgene might have had trophic effects, as no Tg and WT littermates were compared. Collectively, our findings of *MC1R* disruption-induced impairment of αSyn defense and human *MC1R*-mediated rescue strongly support MC1R-specific dopaminergic protection against αSyn.

The ability and specificity of MC1R to protect against αSyn-induced dopaminergic deficits were further demonstrated by BMS‐470,539, a selective MC1R agonist with modest blood–brain barrier permeability, and by locally delivered NDP-MSH, a broader agonist for melanocortin receptors with no brain penetrance, in WT but not *MC1R* mutant mice. Commercially known as Scenesse®, NDP-MSH is pending U.S. Food and Drug Administration review after being approved by the European Medicines Agency to treat patients with erythropoietic protoporphyria. The neuroprotective effects of systemically adminstered NDP-MSH have been reported in models of ischemic stroke, traumatic brain injury, spinal cord injury, and Alzheimer’s disease [15–17]. A more recent study demonstrates the MC1R-dependent neuroprotective effects of intravenously injected NDP-MSH in mouse models of neuroinflammatory disease involving a compromised blood–brain barrier [18]. Activation of MC1R in the brain locally as well as peripherally, perhaps through improvements in the systemic environment, could theoretically protect the nigrostriatal system and may not necessarily exclude each other. While the involvement of peripheral MC1R cannot be ruled out, especially in the context of BMS-470539 neuroprotection, the efficacy of locally administered NDP-MSH suggests that CNS MCIR has protective functions, which is strongly supported by the similar protective effects observed in primary neuronal cultures. Therefore, our study reveals that neuronal MC1R functions as a protective signaling inducer against αSyn neurotoxicity. Further differentiating peripheral versus CNS MC1R actions using cell- or tissue-specific knock-out (e.g., in tyrosinase- and/or TH- expressing cells) in addition to global *MC1R*^e/e^ would be important for further target validation and drug development and for gaining a better understanding of the mechanisms underlying MC1R-mediated dopaminergic defense.

αSyn, especially in its oligomeric form, induces neuroinflammation and oxidative stress, which may contribute to neurodegeneration in PD [48, 57]. We found that exacerbation and protection of αSyn dopaminergic neurotoxicity by genetic and pharmacological MC1R manipulations were accompanied by altered pro-inflammatory cytokines and microglia activation status in the SN. Immune cells including microphages, monocytes, and endothelial cells express MC1R [12, 13]. Expression of MC1R has been reported in a human microglial cell line [58] but not in rat primary microglia [59]. We could not detect MC1R in microglial cells by immunofluorescence double-staining, suggesting that microglial responses may be mediated by neuronal MC1Rs and alterations in neuronal activities. Together with the reported MC1R-dependent protective effect of NDP-MSH against neuroinflammation [18], our findings further support the broad role of MC1R in immunomodulation and inflammation, not only in the periphery [12] but also in the CNS. Despite the previously reported expression of MC1R in a human astrocyte cell line and suggested glial cell MC1R-mediated inhibition of TNF-*α* by *α*-MSH in a mouse model of brain inflammation [58, 60, 61], our double-labeling showed rare colocalization of MC1R and GFAP. MC1R manipulation was not associated with the gliosis that we and others have observed following αSyn overexpression, at least at the time points analyzed.

Additionally, given the predominant transduction of αSyn and MC1R expression in neurons, the altered inflammation and oxidative damage in the ventral midbrain were likely effects of altered Nrf2 signaling in neurons in response to MC1R manipulation. Nrf2, known as a master regulator of the immune system and oxidative stress, was shown to be regulated by MC1R, with impaired induction and activation following αSyn expression in *MC1R*^e/e^ mice; and conversely, with enhanced induction and activation following genetic and pharmacological activation of MC1R in vivo. In cellular models of αSyn pathologies, including primary neuron cultures, the role of Nrf2 was indispensable for the counteractions of MC1R genetic and pharmacological activation. Nrf2 is tightly regulated at multiple levels including transcriptional, post-transcriptional, and post-translational regulation, most importantly in the cytoplasm by its primary negative regulator Kelch-like ECH-associated protein 1 (Keap1) through the canonical and non-canonical mechanisms [62]. Disassociation from Keap1 renders Nrf2 stabilization and translocation into the nucleus, where it activates transcription of the target genes. MC1R activation by α-MSH has been shown to induce Nrf2 mRNA in human skin [37]. In addition, MC1R activates PI3K pathway [63, 64] and PI3K activation in vivo increases Nrf2 mRNA and abundance of Nrf2 protein in nuclear extracts in the liver [65]. A silico promoter analysis of the human *Nrf2* gene identified putative binding site for CREB [37]. The cAMP pathway mediates MC1R signaling [50, 66]. Genome-wide location analysis indicated *Nrf2* as a CREB binding positive gene [51], supporting CREB on the *Nrf2* promoter. We demonstrated that MC1R overexpression in αSyn expressing HEK293T cells increased Nrf2 mRNA and protein as well as nuclear Nrf2, suggesting that Nrf2 activation and target gene HO-1 expression were likely the results of de novo Nrf2 production and nuclear accumulation. Indeed, ChIP assay demonstrated increased CREB binding to the *Nrf2* promoter following MC1R activation, further supporting transcriptional activation of Nrf2 by MC1R through CREB. Although MC1R overexpression in HEK293T cells appeared to induce CREB binding, mRNA, protein, and nuclear translocation of Nrf2 to the comparable extend, other mechanisms may still contribute to MC1R activation of Nrf2 in the context of αSyn toxicity. Nevertheless, these results provide direct evidence that Nrf2 is a downstream mediator of the protective effects of MC1R activation. Our findings also support the ability of Nrf2 in neurons to be responsive [53], both in vivo and in vitro, to MC1R manipulation in the context of αSyn toxicity. Nrf2 is altered in Parkinson’s and related neurodegenerative diseases, and Nrf2 activators have progressed to active clinical practice or development as neurotherapeutics [4, 53, 67–69]. Although we were not able to assess cytoplasmic *vs.* nuclear Nrf2 due to limited tissue amount, our results indicated reduced Nrf2 in PD postmortem SN that was at the margin of statistical significance. Collectively, these findings provide not only mechanistic insight into MC1R defense against αSyn pathologies but also therapeutic implications for the possible use of Nrf2 activators in PD patients, especially those carrying partial or complete loss-of-function MC1R variants.

The translational significance of our findings is further highlighted by the presence of MC1R in dopaminergic neurons in the human SN and the suggestion of reduced MC1R expression at the tissue level in PD patients *vs.* controls. MC1R immunoreactivity in periaqueductal gray neurons is reported in fixed human brain sections [70]. Mykicki et al. described the expression of MC1R in human neuronal cells differentiated from a progenitor cell line and in neurons from postmortem human brain tissues [18]. The same study reported reduced brain MC1R expression in multiple sclerosis patients, although no brain regions or neuron types were specified [18]. The remarkable overlap of MC1R and TH in the SN observed in our study suggests primary, if not exclusive, dopaminergic neuronal functions of MC1R within the SN. Notably, MC1R did not appear to be reduced at the cellular level in surviving dopaminergic neurons. MC1R expression was unaltered in the ventral midbrain in AAV αSyn mice at an earlier timepoint when dopaminergic neuron loss was not yet significant. Although it remains to be determined whether loss of MC1R at the tissue level in PD patients precedes loss of dopaminergic neurons, the evidence presented by this and other studies supports that MC1R is involved mechanistically in the pathophysiology of PD and is a promising therapeutic target for PD and related disorders. The findings encourage development of CNS-penetrant MC1R agonists as well as potential repurposing of existing, primarily peripherally acting MC1R agonists, as candidate disease-modifying therapy for PD. Given the links among MC1R loss of function, red hair, melanoma, and PD, our findings also provide evidence of a possible MC1R basis for the well-established link between PD and melanoma. The similar protection observed in vitro in cells that are considered non-pigmented and lack pigmentation machinery, including HEK293T cells and primary cortical neurons, supports non-pigmentary pathways of MC1R actions. Further studies are needed to determine whether *MC1R* as the key pigmentation gene acts through pigmentation and whether neuromelanin or melanin in the periphery is involved in MC1R neuroprotection in vivo.

## Conclusions

By using genetic and pharmacological approaches, our study demonstrated in multiple in vivo and in vitro models that MC1R loss-of-function leads to exacerbated PD-associated αSyn pathologies. Conversely MC1R activation is neuroprotective against αSyn-induced neurotoxicity. The protective effects of MC1R can be mediated by Nrf2, at least in vitro. Together with the human postmortem tissue data and the previous epidemiological and biological studies from our group and others, these results suggest that MC1R signaling plays a role in neurodegeneration in PD. They provide evidence for MC1R as a therapeutic target and a rationale for development of MC1R-activating strategies for PD.

## Supplementary Information

**Additional file 1. **

## Data Availability

All data generated or analyzed during this study are included in this published article and its supplementary information files.

## Abbreviations

AAV: Adeno-associated virus
BiFC: Bimolecular fluorescence complementation
CBP: CAMP response element binding protein (CREB) binding protein
ChIP: Chromatin immunoprecipitation
CREB: CAMP response element binding protein
GCLC: Glutamate-cysteine ligase subunit C
GFAP: Glial fibrillary acidic protein
HO1: Heme oxygenase 1
HPLC: High-performance liquid chromatography
iba1: Ionized calcium binding adapter molecule 1
ICAM1: Intercellular adhesion molecule 1
IL-6: Interleukin (IL)-6
LDH: Lactate dehydrogenase
MC1R: Melanocortin 1 receptor
NDP-MSH: Nle4,D-Phe7-α-MSH
NQO1: NAD(P)H quinone dehydrogenase 1
Nrf2: Nuclear factor erythroid 2-related factor 2
PD: Parkinson disease
p-αSyn: Phosphorylated αSyn at serine 129
pCREB: Phosphorylated CREB at serine 133
qPCR: Quantitative polymerase chain reaction
scRNA: Scrambled RNA
SN: Substantia nigra
SNpc: SN pars compacta
Tg: Transgenic
TH: Tyrosine hydroxylase
TNF: Tumor necrosis factor
WT: Wild-type
α-MSH: Alpha-melanocyte stimulating hormone
