# Supplementary material for: Melanocortin 1 receptor activation protects against alpha-synuclein pathologies in models of Parkinson’s disease
Source: Mol Neurodegener. 2022 Feb 23;17:16. doi: 10.1186/s13024-022-00520-4 (PMC8867846; doi:10.1186/s13024-022-00520-4)
Supplement: Supplementary file 1 — Additional file 1. [file 13024_2022_520_MOESM1_ESM.docx]

**Supplementary Information for**

**Melanocortin 1 receptor activation protects against alpha-synuclein pathologies in models of Parkinson’s Disease**

Waijiao Cai^1,2^, Pranay Srivastava^1,7^, Danielle Feng^1^, Yue Lin^1^, Charles R. Vanderburg^1,3^, Yuehang Xu^1^, Pamela Mclean^4^, Matthew P. Frosch^1,3,5^, David E. Fisher^6^, Michael A. Schwarzschild^1,7^, Xiqun Chen^1,7*^

^1^MassGeneral Institute for Neurodegenerative Disease, Department of Neurology, Massachusetts General Hospital, Harvard Medical School, Boston, USA.

^2^Department of Integrative Medicine, HuaShan Hospital, Institutes of Integrative Medicine, Fudan University, Shanghai, China.

^3^Harvard NeuroDiscovery Advanced Tissue Resource Center, Massachusetts General Hospital, Harvard Medical School, Boston, USA.

^4^Mayo Clinic, Jacksonville, Florida, USA.

^5^Neuropathology Service, Massachusetts General Hospital, Harvard Medical School, Boston, USA.

^6^Cutaneous Biology Research Center, Department of Dermatology, Massachusetts General Hospital, Harvard Medical School, Boston, USA.

^7^Aligning Science Across Parkinson’s (ASAP) Collaborative Research Network, Chevy Chase, Mayland, USA.


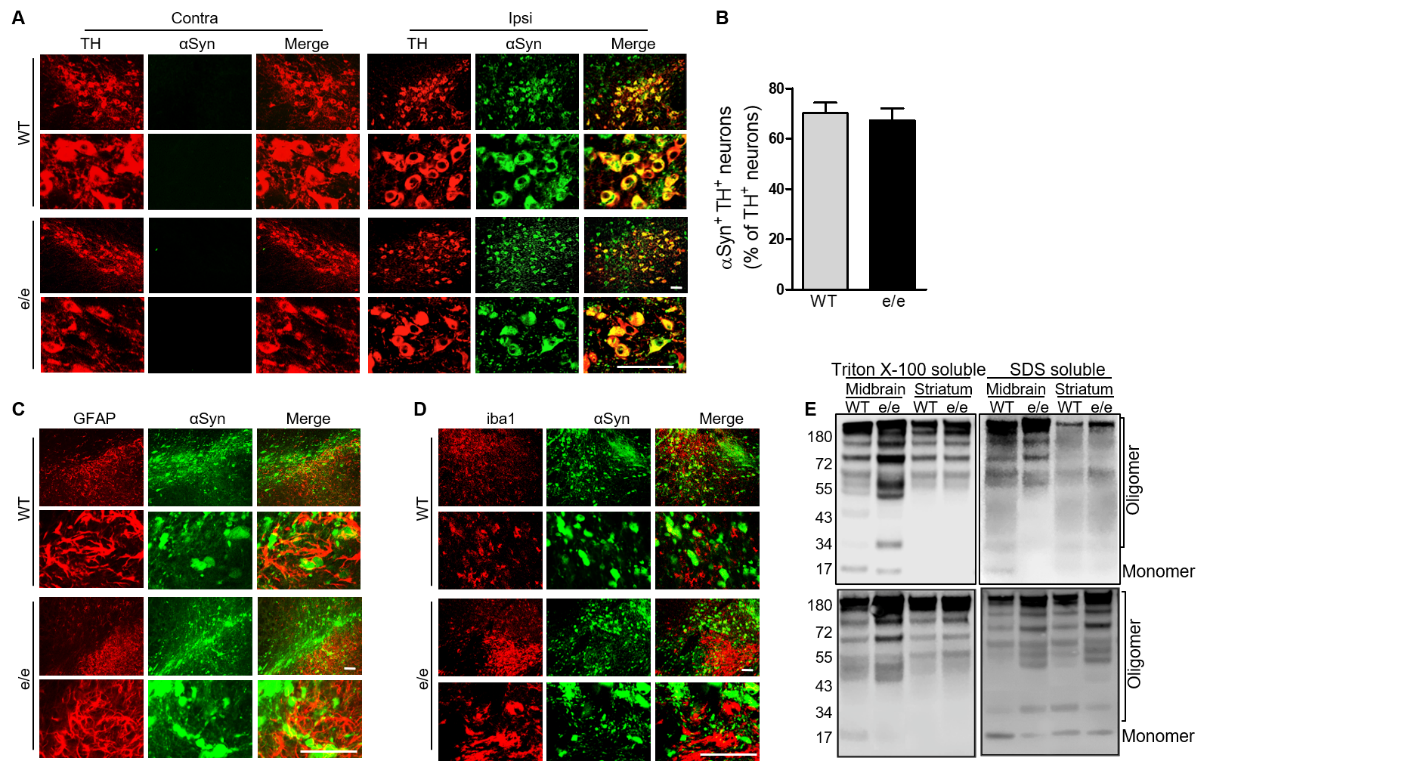


**Fig. S1. Human αSyn transduction in dopaminergic neurons and glial and microglial cells in the SN.** *MC1R* ^e/e^ and WT mice were injected unilaterally with human αSyn AAV into the SN and sacrificed 4 weeks later. (A) Immunofluorescence double-staining for TH and human αSyn. Scale bars, 50 µm. (B) Percentage of αSyn-transduced dopaminergic neurons in the ipsilateral SN. Student’s *t* test. n=5 mice/group. (C) Immunofluorescence double-staining for GFAP and human αSyn on the ipsilateral side. Scale bars, 50 µm. (D) Immunofluorescence double-staining for iba1 and human αSyn on the ipsilateral side. Scale bars, 50 µm. (E) Immunoblot of human αSyn species in Triton X-100-soluble and -insoluble SDS-soluble fractions in ipsilateral ventral midbrain and striatum. n=3 mice/group (sample #2, 3; #1 see Fig. 1A).


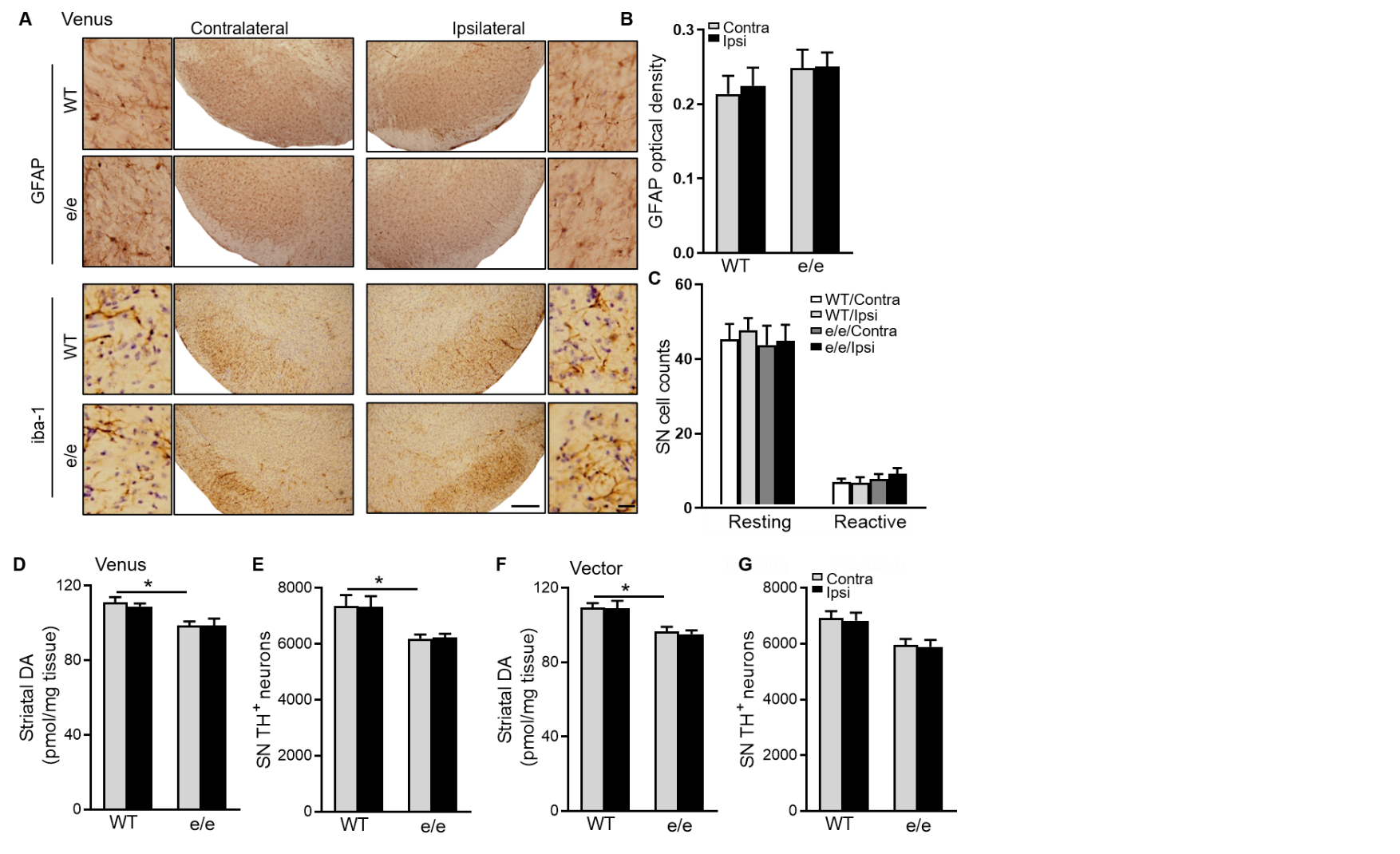


**Fig. S2. Venus or vector did not induce astrogliosis, microgliosis or dopaminergic neuron loss in the SN.***MC1R*^e/e^and WT mice were injected unilaterally with venus or vector into the SN and sacrificed 12 weeks later. (A) GFAP or iba-1 immunostaining in the SN. Scale bars, lower magnification 100 µm, higher magnification 10 µm. (B) quantification of integrated optical density of GFAP in the SN. Two-way ANOVA followed by Tukey’s post hoc test. n=4 mice/group. (C) Morphological classification and quantification of iba1-positive cells in the SN. Two-way ANOVA followed by Tukey’s post hoc test. n=4 mice/group. (D) Striatal dopamine content and (E) stereological quantification of TH-positive in the SN with venus injection. Two-way ANOVA followed by Tukey’s post hoc test. n=6 mice/group. (F) Striatal dopamine content and (G) stereological quantification of TH-positive in the SN with empty vector injection. Two-way ANOVA followed by Tukey’s post hoc test. n=6 mice/group. **P*<0.05.

**
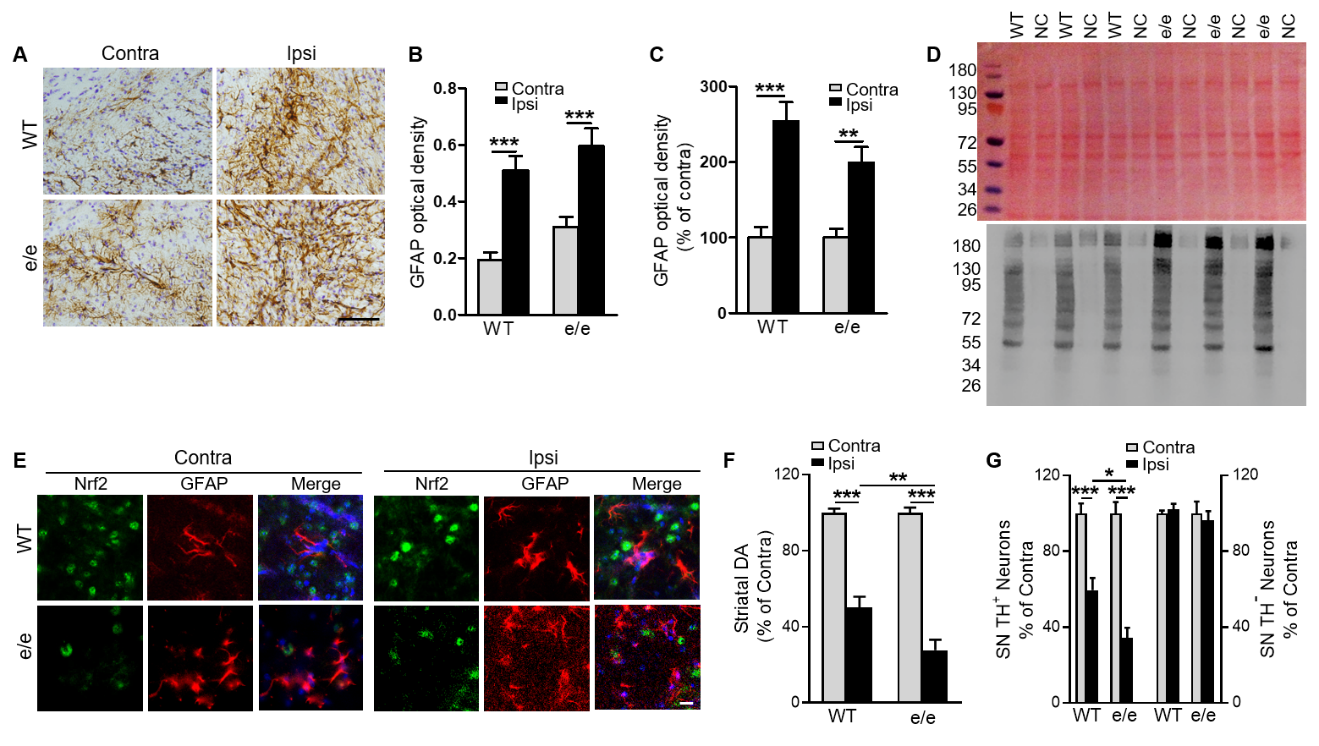
**

**Fig. S3.** **αSyn-induced astrogliosis, Nrf2 expression in astrocytes, and exacerbated dopaminergic neurotoxicity in** *MC1R*^e/e^ **mice***. MC1R*^e/e^ and WT mice were injected unilaterally with human WT αSyn AAV into the SN and sacrificed 12 weeks later. (A) GFAP staining in the SN and (B) quantification of optical density in original values or (C) normalized to contralateral values. Measurements were normalized by dividing values by the mean of the contralateral side and multiplying by 100. Two-way ANOVA followed by Tukey’s post hoc test. n=6 mice/group. Scale bar, 25 µm. (D) Oxyblot for protein carbonyls and the corresponding Ponceau S staining in the ipsilateral ventral midbrain (all samples). NC, samples incubated with negative control solution instead of 2,4-dinitrophenylhydrazine (DNPH) solution. (E) Immunofluorescence double-staining of Nrf2 and GFAP 4 weeks post-AAV injection. Scale bar, 10 µm. *MC1R*^e/e^ and WT mice were injected unilaterally with human WT αSyn AAV into the SN and sacrificed 16 weeks later. (F) Striatal dopamine content and (G) stereological quantification of TH-positive and -negative cells in the SN. Measurements were normalized by dividing values by the mean of the contralateral side and multiplying by 100. Two-way ANOVA followed by Tukey’s post hoc test. n=12 mice/group. **P*<0.05, ***P*<0.01, ****P*<0.001.

**
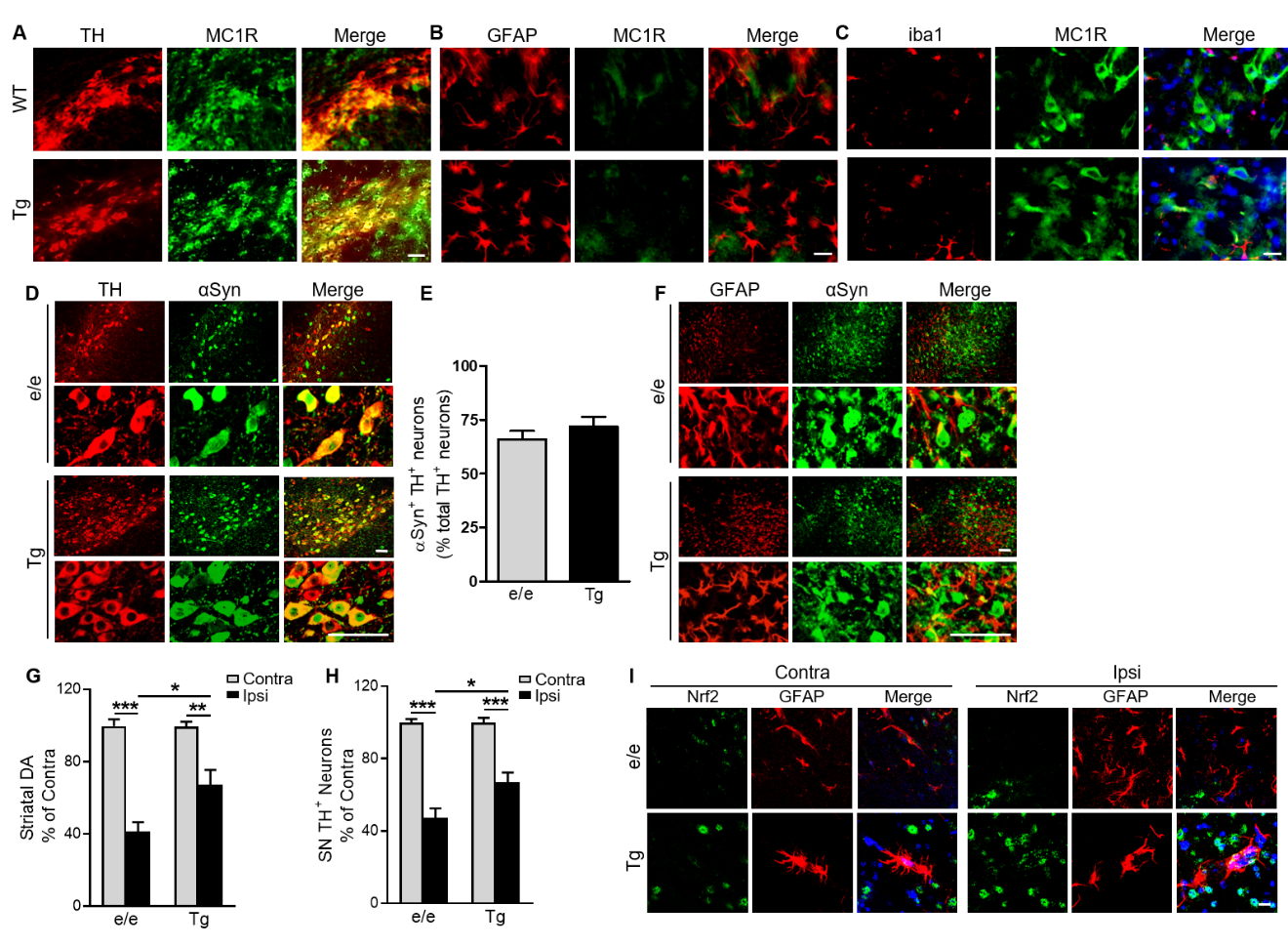
Fig. S4. Tg *MC1R* expression, αSyn transduction, Nrf2 expression in the SN, and dopaminergic neurotoxicity in *MC1R*^e/e^ Tg mice.** (A) Immunofluorescence double-staining for TH and MC1R. Scale bar, 50 µm. (B) Immunofluorescence double-staining for GFAP and MC1R. Scale bar, 10 µm. (C) Immunofluorescence double-staining for iba1 and MC1R. Scale bar, 10 µm. *MC1R*^e/e^Tg and *MC1R*^e/e^ mice were injected unilaterally with human αSyn AAV into the SN. (D) Immunofluorescence double-staining for TH and human αSyn and (E) percentage of αSyn-transduced dopaminergic neurons 4 weeks post-AAV injection. Student’s *t* test. n=4 mice/ group. Scale bars, 50 µm. (F) Immunofluorescence double-staining of GFAP and human αSyn 4 weeks post-AAV injection. Scale bars, 50 µm. *MC1R*^e/e^Tg and *MC1R*^e/e^ mice were injected unilaterally with human WT αSyn AAV into the SN. (G) Striatal dopamine content and (H) stereological quantification of TH-positive and negative cells 16 weeks post-AAV injection. Measurements were normalized by dividing values by the mean of the contralateral side and multiplying by 100. Two-way ANOVA followed by Tukey’s post hoc test. n=6-7 mice/group. **P*<0.05, ***P*<0.01, ****P*<0.001. (I) Immunofluorescence double-staining of Nrf2 and GFAP 4 weeks post-AAV injection. Scale bar, 10 µm.

**
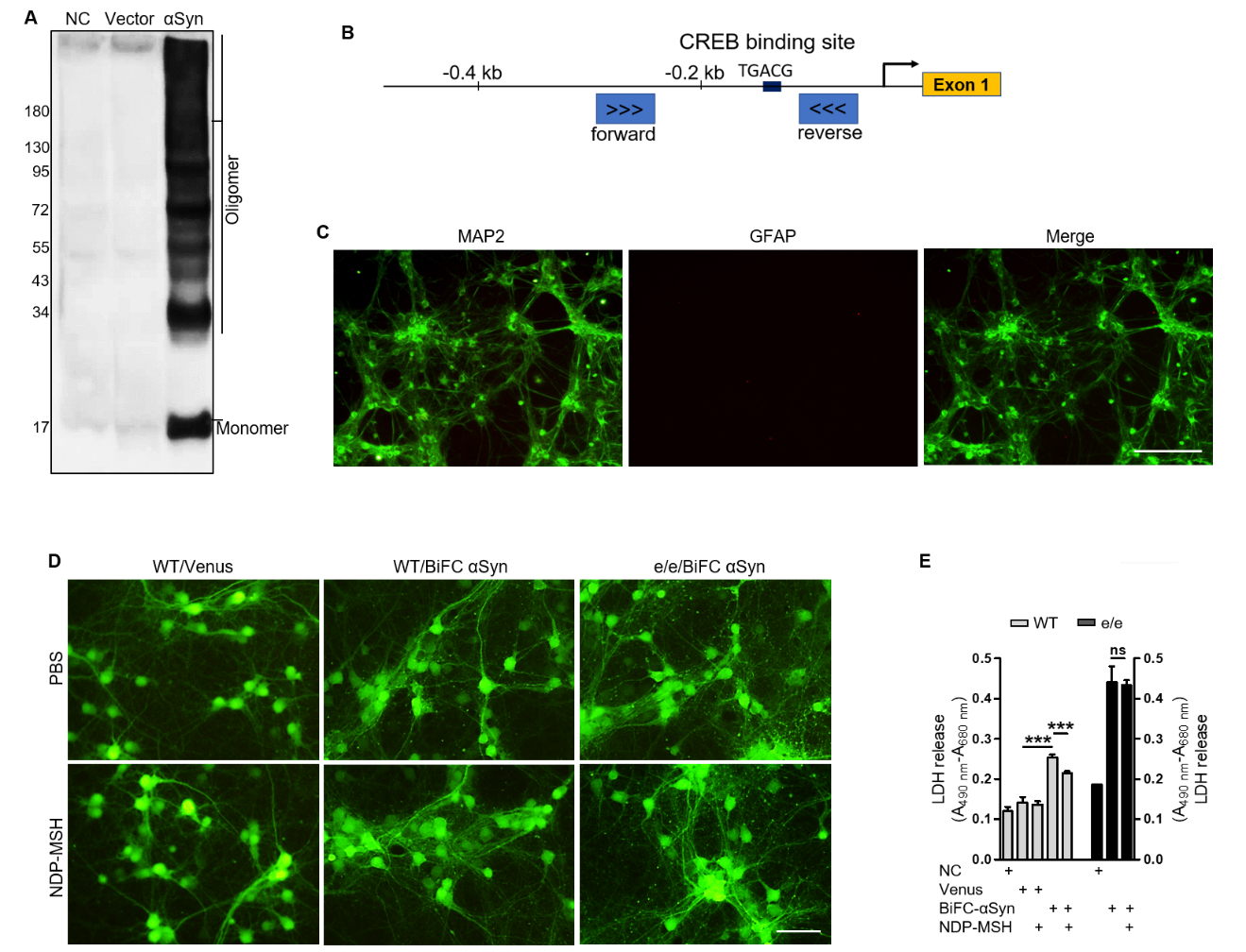
Fig. S5. αSyn species in HEK293T cells overexpressing human WT αSyn; ChIP-qPCR primers; characterization of primary cortical neurons and protection of MC1R activation against αSyn cytotoxicity.** (A) Immunoblot for oligomeric and monomeric αSyn in HEK293T cells transfected with human WT αSyn or control vector. NC: non-transfected control cells. (B) ChIP-qPCR primers for CREB binding site in the *Nrf2* promoter. (C) MAP2 and GFAP staining in WT primary cortical neurons at DIV5. Scale bar, 25 µm. (D) αSyn oligomerization visualized by fluorescence at DIV9 in WT or *MC1R*^e/e^ cortical neurons transduced with BiFC αSyn AAV or control venus AAV. Scale bar, 25 µm. (E) LDH release at DIV12 in WT or *MC1R*^e/e^ cortical neurons transduced with BiFC αSyn or venus AAV and treated with NDP-MSH. One-way ANOVA followed by Tukey’s post hoc test. ****P*<0.001. ns=not statistically significant.

**
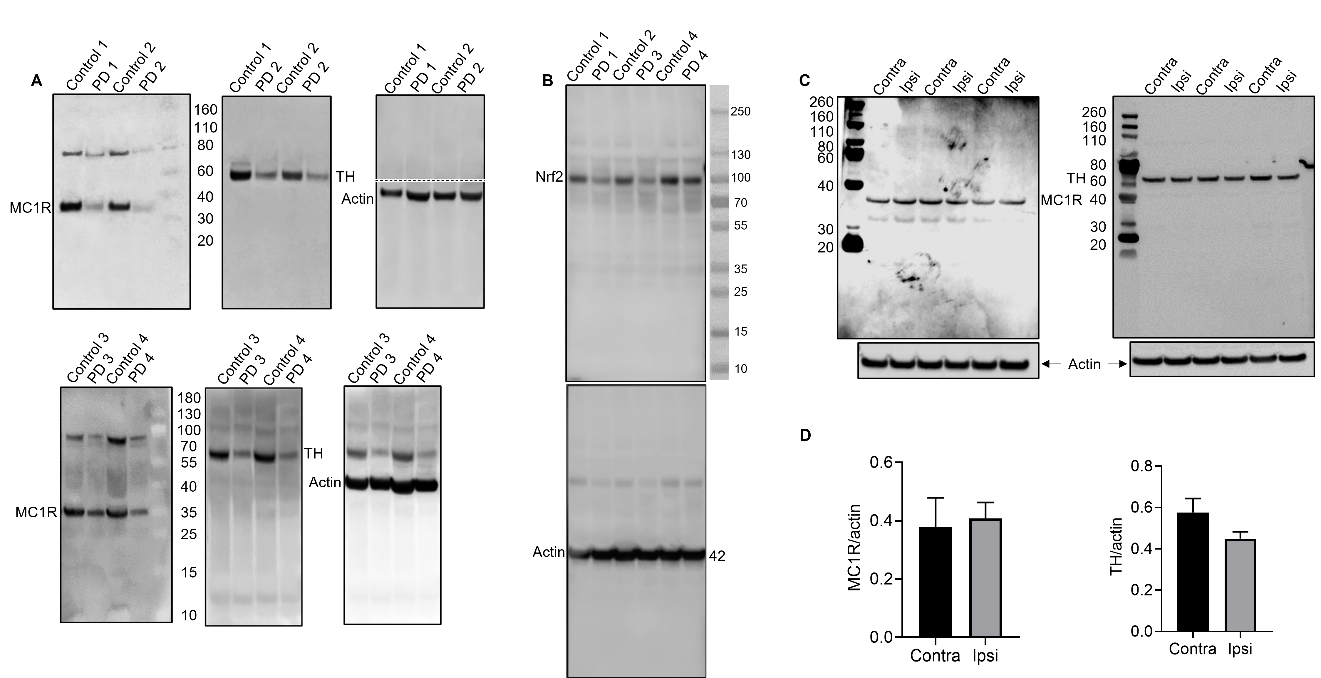
Fig. S6. MC1R expression in PD postmortem SN and in αSyn mouse model of PD.** (A) Full-lane immunoblots corresponding to cropped blots in Fig. 6E for MC1R, TH, and actin using SN tissue from control individuals and PD patients. Actin blot of control 1&2 and PD 1&2 was cut as indicated by the dashed line and probed as a whole. (B) Full-lane immunoblots corresponding to cropped blots in Fig. 6G for Nrf2 and actin using SN tissue from control individuals and PD patients. (C) C57Bl/6J mice were injected with αSyn AAV into the SN and sacrificed 6 weeks later. Immunoblot for MC1R and TH using ventral midbrain tissue from ipsilateral injected (ipsi) side and contralateral non-injected (contra) side. (D) quantification of MC1R and TH band density. Two-tail Student’s *t*-test. n=3 mice/group.


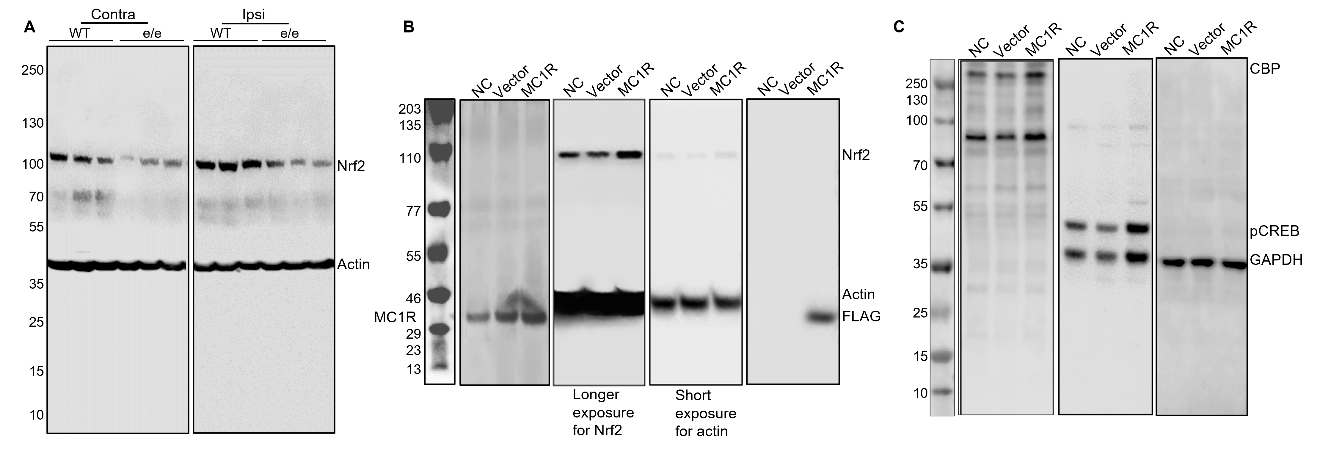


**Fig. S7. Full-lane immunoblots.** (A) Full-lane immunoblots corresponding to cropped blots in Fig. 2F for Nrf2 and actin. (B) Full-lane immunoblots corresponding to cropped blots in Fig. 5A for Nrf2, MC1R, FLAG, and actin. (C) Full-lane immunoblots corresponding to cropped blots in Fig. 5C for CBP, pCREB, and GAPDH.

| **Supplementary Table 1: Primers used for qPCR** | |
| --- | --- |
| **Gene name** | **Primer sequence** |
| Mouse |  |
| *IL-6* | F: TAGTCCTTCCTACCCCAATTTCC |
|  | R: TTGGTCCTTAGCCACTCCTTC |
| *TNF-α* | F: CCCTCACACTCAGATCATCTTCT |
|  | R: GCTACGACGTGGGCTACAG |
| *ICAM1* | F: TCAGTGGCTGAAAGATGAGC |
|  | R: CGGAAACGAATACACGGTGA |
| *IL-1α* | F: GACAGGGAACTTAGGGAGCA |
|  | R: TTGGCCATCTTGATTTCAGAGT |
| *HO-1* | F: GCTACCTGGGTGACCTCTCA |
|  | R: GCAGCTCCTCAAACAGCTCAAT |
| *NQO-1* | F: CCTTTCCAGAATAAGAAGACC |
|  | R: AATGCTGTAAACCAGTTGAG |
| *GCLC* | F: CTATCTGCCCAATTGTTATGG |
|  | R: ACAGGTAGCTATCTATTGAGTC |
| *GCLM* | F: GCACAGGTAAAACCCAATAG |
|  | R: TTAGCAAAGGCAGTCAAATC |
| GAPDH | F: CATGGCCTTCCGTGTTCCTA |
|  | R: CCTGCTTCACCACCTTCTTGAT |
| Human |  |
| *Nrf2* | F: AGTGGATCTGCCAACTACTC |
|  | R: CATCTACAAACGGGAATGTCTG |
| *HO-1* | F: ACTGCGTTCCTGCTCAACATC |
|  | R: GCTCTGGTCCTTGGTGTCATG |
| *GAPDH* | F: CATGAGAAGTATGACAACAGCCT |
|  | R: AGTCCTTCCACGATACCAAAGT |
